# Socioeconomic position and childhood-adolescent weight status in rich countries: a systematic review, 1990–2013

**DOI:** 10.1186/s12887-015-0443-3

**Published:** 2015-09-21

**Authors:** Laura Barriuso, Estrella Miqueleiz, Romana Albaladejo, Rosa Villanueva, Juana M. Santos, Enrique Regidor

**Affiliations:** 1 Instituto de Salud Pública y Laboral de Navarra, Pamplona, Spain; 2 Department of Sociology, Universidad Pública de Navarra, Campus de Arrosadía, s/n, 31006 Pamplona, Spain; 3 Department of Preventive Medicine and Public Health, Faculty of Medicine, Universidad Complutense de Madrid, Madrid, Spain; 4 Instituto de Investigación Sanitaria del Hospital Clínico San Carlos (IdISSC), Madrid, Spain; 5 CIBER Epidemiología y Salud Pública (CIBERESP), Madrid, Spain

**Keywords:** Childhood obesity, Socioeconomic position, Systematic review, Rich countries

## Abstract

**Background:**

Childhood obesity is a major problem in rich countries due to its high prevalence and its harmful health consequences. An exploratory analysis conducted in the PubMed database highlighted that the number of papers published on the relationship between socioeconomic position (SEP) and childhood-adolescent weight status had risen substantially with respect to an earlier review which had covered the period 1990–2005.

**Methods:**

To describe the findings on the relationship between SEP and childhood-adolescent weight status in papers published in rich countries from 1990 through 2013, studies were identified in the following databases: PubMed; Web of Knowledge (WOK); PsycINFO; Global Health; and Embase. We included observational studies from the 27 richest OECD countries, which covered study populations aged 0 to 21 years, and used parental education, income and/or occupation as family SEP indicators. A total of 158 papers met the inclusion criteria and reported 134 bivariable and 90 multivariable analyses.

**Results:**

Examination of the results yielded by the bivariable analyses showed that 60.4 % of studies found an inverse relationship, 18.7 % of studies did not found relationship, and 20.9 % of studies found a relationship that varied depending on another variable, such as age, sex or ethnic group; the corresponding percentages in the multivariable analyses were 51.1, 20.0 and 27.8 %, respectively. Furthermore, 1.1 % found a positive relationship.

**Conclusion:**

The relationship between SEP and childhood-adolescent weight status in rich countries is predominantly inverse and the positive relationship almost has disappeared. The SEP indicator that yields the highest proportion of inverse relationships is parents’ education. The proportion of inverse relationships is higher when the weight status is reported by parents instead using objective measurements.

**Electronic supplementary material:**

The online version of this article (doi:10.1186/s12887-015-0443-3) contains supplementary material, which is available to authorized users.

## Background

In rich countries, childhood obesity is a public health problem of the first order due to its high prevalence and short- and long-term health repercussions [1–5]. Childhood obesity is not only an important risk factor for childhood/adolescent diseases, such as diabetes mellitus, sleep apnea, asthma, precocious puberty and psychological disorders [5–7], but because a high percentage of obese children become obese adults. Among the factors that increase the likelihood of childhood obesity are the social determinants such as the SEP [8].

The relationship between SEP and body weight was already suggested in the latter part of the 19^th^ century. In 1889, Veblen considered that slimness was an ideal of feminine beauty, indicative of the social status pertaining to the emerging leisure social class of the age [9]. Several decades had to elapse before the relationship between SEP and weight status came to be examined. Indeed, it was a century later, in 1989, when Sobal and Stunkard published an extensive bibliographic review on the influence exerted by SEP on obesity among children and adults in developed and developing countries [10]. They analyzed a total of 144 studies published in the preceding 40 years and concluded that the relationship varied according to subject’s age and sex, and countries’ degree of development. In developed countries, they observed a clear inverse relationship between SEP and obesity in women -in that the highest and lowest proportions of obesity were observed in women of low and high SEP, respectively- and an inconsistent relationship in men, boys and girls. For example, 35 papers published between 1941 and 1986 made reference to the relationship between SEP and obesity in children and adolescents. In boys an inverse relationship was observed in 32 % of studies, a positive relationship -the highest and lowest proportions of obesity in high and low SEP, respectively- in 26 % and an absence of relationship in 41 %; in girls an inverse relationship was observed in 40 % of studies, a positive relationship in 25 %s and an absence of relationship in 35 %. In developing countries, however, an intense positive relationship was observed in women, men, boys and girls.

In 2008, Shrewsbury and Wardle published another systematic review focusing exclusively on the relationship between SEP and childhood adiposity in developed countries [11]. It included 45 studies, published from January 1990 through December 2005, which had been conducted on populations aged 5 to 18 years, using family and neighborhood SEP indicators. Again, the relationship found was inconsistent −42 % inverse relationship, 27 % absence of relationship, 31 % inconclusive- Only one positive relationship between PES and obesity, in girls in an adjusted analysis, was observed. A comparison with the results of the previous review highlighted a change in the pattern of this relationship, i.e., while studies reporting a positive relationship had disappeared, the percentage of studies displaying an inverse relationship had risen.

Faced with the prospect of new findings, an exploratory bibliographic search was made in The National Library of Medicine’s PubMed to bring the situation up to date, and revealed a sharp rise in scientific activity in this area from 2005. Hence, the idea of updating the systematic review and extending it to other databases was deemed to be of interest. An increase in the percentage of studies whose results show an inverse relationship would support intervention strategies that seek to reduce childhood obesity, directing the focus to the lower socioeconomic groups. Specifically, the aim of this study was to report the findings of a systematic review of epidemiologic evidence of the association between family SEP and weight status in the child-adolescent population in high income countries, across the period 1990–2013.

## Methods

The dependent variable used for the purposes of this systematic review was childhood-adolescent weight status, measured either by reference to the presence of excess weight (overweight and/or obesity) or some anthropometric parameter. To identify potentially relevant publications, we conducted a bibliographic search in available databases having the greatest scientific impact on the medical and social fields. Accordingly, the following five databases were analyzed: The National Library of Medicine’s PubMed; Web of Knowledge (WoK); PsycINFO; Global Health; and Excerpta Medica Database (Embase). In the selection of the search strategy we follow the recommendations of Pettigrew and Roberts [12]. Because we used multiple bibliographic databases and the studies in some databases do not have structured abstract and keywords, we decided to use the search terms as major topics in each database, in order that the search be more comprehensive to identify all relevant studies. Previously we got a sample of studies from each database that met the inclusion criteria we discuss below. In this way we get to find out the major topics terms that were used in each database to search for potential relevant articles. The main characteristics of the search strategy used are shown in Table 1.

**Table 1:** Bibliographic search strategy

Database	Search terms
PubMed	(“Obesity” (MeSH Major Topic) OR “overweight” (MeSH Major Topic) OR “adiposity” (MeSH Major Topic) OR “body mass index” (MeSH Major Topic)) AND (“social class” (MeSH Major Topic) OR “occupations” (MeSH Major Topic) OR “employment” (MeSH Major Topic)).
Web of Knowledge (WoK)	Topic “Childhood obesity” AND Topic “social class”.
PsycINFO	1. (Socioeconomic and Status and Adiposity and Childhood).mp. [mp = title, abstract, heading word, table of contents, key concepts]; 2. socioeconomic status/ or “income (economic)”/ or poverty/; 3. from 1 keep 1–8; 4. body fat/ or body weight/; 5. exp Obesity/ or obesity.mp.; 6. Adiposity.mp.; 7. Childhood.mp.; 8. children.mp.; 9. 4 or 5 or 6; 10. 7 or 8; 11. 2 and 9 and 10; 12. Socioeconomic and Status and Adiposity and Childhood; 13. limit 12 to (100 childhood and five stars and last 20 years); 14. from 11 keep 1–90.
Global Health	1. Socioeconomic and Status and Adiposity and Childhood; 2. limit 1 to last 24 years; 3. exp obesity/; 4. socioeconomic status/ or social status/ or economic sociology/ or living conditions/; 5. children/; 6. Childhood.mp.; 7. 5 or 6; 8. 3 and 4 and 7.
Embase	1. Socioeconomic and Status and Adiposity and Childhood; 2. (Socioeconomic and Status and Adiposity and Childhood).mp. [mp = title, abstract, subject headings, heading word, drug trade name, original title, device manufacturer, drug manufacturer]; 3. social status/; 4. from 3 keep 1–27; 5. obesity/di, ep, et, pc, si [Diagnosis, Epidemiology, Etiology, Prevention, Side Effect]; 6. child/; 7. 3 and 5 and 6.

The inclusion criteria of the papers were: a) published from January 1, 1990 through December 31, 2013; b) in English or Spanish; c) study participants having an age range of 0 to 21 years; d) observational studies; e) use of parental education, income, and/or occupation as the family SEP indicator; and, f) conducted in a “high income country”, defined as any of members of the Organization for Economic Cooperation and Development (OECD) having a gross domestic product per head (US $, current PPPs) higher than 25,000 dollars, according to the International Monetary Fund’s figures for 2010. These countries were Australia, Austria, Belgium, Canada, Czech Republic, Denmark, Finland, France, Germany, Greece, Holland, Iceland, Ireland, Israel, Italy, Japan, Korea, Luxembourg, New Zealand, Norway, Portugal, Slovenia, Spain, Sweden, Switzerland, United Kingdom and the USA.

The exclusion criteria ruled out any paper that: a) reported an intervention study; b) used race-ethnic group as a SEP indicator; or c) only included area-level SEP index. We decided to exclude the SEP of the area due to the heterogeneity of the level of aggregation of the areas -sections census, districts, provinces, regions, countries-, in these studies, the variety of area-level SEP index and the variety of measures of individual SEP used by researchers in these studies to control for residual confounding of the association between area-level SEP index and health outcome [13].

### Methods Data extraction and quality assessment

Potentially relevant papers were selected by screening the titles, abstract and the entire articles through the database searches. Two authors (LB and JMS) independently conducted this screening. Disagreement about eligibility between the reviewers was solved through discussion with a third author (ER). Two authors (LB and EM) extracted the data from the included studies using a pilot data extraction form. Data relating to sample size, sex and age of study subjects, date of the study, weight status outcomes (objectively measured, parent-report, cut-off points), familiar socioeconomic position and findings by familiar socioeconomic position were extracted.

All studies were quality assessed by one author (RA) and checked by another (RV). Since there are a variety of checklist and scales to evaluate the quality of observational studies and they differ by content, format, validity and applicability to different studies [14, 15], none of the proposed instruments was used. In any case, each study was assessed using the following item: clear definition of the objective and the study population, justification of the sample size and representativeness of the same, clear definition of the independent and dependent variables, measuring variables in all study subjects or absence of information from these variables in a proportion of subjects and assessment of potential confounders in multivariate analysis. Also, we followed PRISMA guidelines to report the systematic review.

### Data analysis

We have not performed formal meta-analysis as the necessary conditions of comparability of exposures, together with homogeneity of association direction and strength [16], are not met. Results are presented as a narrative synthesis. This paper reports the results of bivariable and multivariable analyses. Whereas the bivariable analyses show the results for the SEP/weight status relationship obtained on the basis of crude analyses or analyses adjusted for age and/or sex, the multivariable analyses show the results of analyses that included other variables of adjustment. Results were classified into “inverse relationship”, “positive relationship” or “absence of relationship”. A relationship was defined as “inverse” when the measure of weight status displayed the highest and lowest magnitudes in participants of low and high SEP, respectively, and in addition, when there was a statistically significant difference between them, and/or the *p*-value for linear trend across the different SEP categories was statistically significant. A relationship was defined as “positive” when the measure of weight status displayed the lowest and highest magnitudes in participants of low and high SEP, respectively, and in addition, when there was a statistically significant difference between them, and/or the *p*-value for linear trend across the different SEP categories was statistically significant. In all cases, a *p*-value of less than 0.05 was taken as the criterion of statistical significance. An “absence of relationship” was deemed to exist when there was no statistically significant relationship between the indicator of weight status and SEP. In any case when a paper reported different results in different population groups analyzed, in different study periods, or according to the type of dependent variable analyzed, this was described as a relationship that “varies depending on another variable”. In order to render the presentation of the findings as uniform as possible, we calculated the *odds ratios* in those cases in which the articles only showed frequency measures, either by taking the highest SEP category as reference, or alternatively by re-estimating the measure of association shown if the reference category used was the lowest SEP. In cases where papers showed no results tables, we included those that the authors cited literally in the text.

## Results

**Fig. 1:**
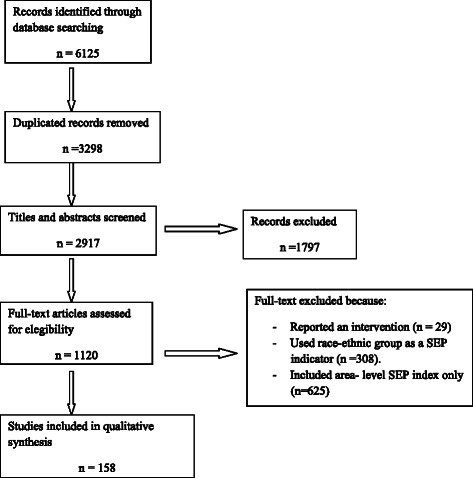
PRISMA flow diagram

The electronic search produced 6215 references. After removing duplicate references titles and abstract were screened. One thousand and one hundred twenty full-text outputs were assessed; 158 outputs were included and 962 were excluded (Fig. 1):671 because they did not meet some of the inclusion criteria, 29 because they were intervention studies, 204 because they used race-ethnic and 58 because they only used area-level SEP index. The 158 papers that fulfilled the inclusion criteria are shown in Additional file 1: Table S1. The studies came from the following countries: 48 from the USA [17–64]; 22 from the United Kingdom [65–86]; 18 from Germany [87–104]; 11 from Australia [105–115]; eight from France [116–123]; seven from Spain [124–130]; six from Canada [131–136]; five from Sweden [137–141], Greece [142–146] and Holland [147–151]; four from Belgium [152–155] and Italy [156–159]; two from Finland [160, 161], Portugal [162, 163], Denmark [164, 165] and Ireland [166, 167]; one from several countries [168] and one each from the Czech Republic [169], Iceland [170], Israel [171], Korea [172], Norway [173] and Switzerland [174].

The studies analyzed samples of boys and girls, except in three cases in which only girls were included [41, 48, 57]. Sample size varied widely, ranging from 77 to 90,808 participants. All the studies analyzed participants aged 0 to 21 years, except one in which the age range was 2 to 24 years: this was nonetheless included in our review because it did not present the data broken down by age [126]. Twenty one studies examined individuals of a single age [20, 42, 51, 65, 69, 70, 72, 73, 80, 88, 106, 131, 132, 137–140, 149, 154, 166, 170]. Four cases [25–27, 134] did not mention the individuals” ages but referred instead to their current school grade. In three instances, papers reported the same results but, seeing as the authors’ objectives were different [46, 60, 61], they were analyzed separately.

Table 2 shows the number of papers according to period of publication, region of origin, SEP indicator, method for collecting anthropometric data, and criterion used for defining weight status. The earliest paper analyzed had been published in 1992 [82], and over half of the papers appeared in the period 2006–2013. All were published in English, except two which were published in Spanish [126, 130]. Just over half the papers came from Europe and one third from North America. While 28.5 % of the papers used the father’s and/or mother’s education as the SEP indicator, nearly half the papers used various SEP indicators. The measurement of weight most frequently used was Body Mass Index (BMI), with weight and height being measured by the researchers themselves in 78.5 % of the papers. In 63.3 % of papers the references used for the definition of excess weight were tables or cut-off points of a national or supranational nature. In 37 % of the studies it was used internationally accepted cut-off points defined by the International Obesity Task Force (IOTF) [175], and from 2001 onwards, almost half the papers published used this definition of excess weight. In 20 % of the studies it was used the CDC cut-off points, although most of these studies come from USA.

**Table 2:** Number of papers found on the relationship between family socioeconomic position and weight status in the child-adolescent population, according to different classification criteria: period 1990–2013

	Number	Percent
Total	158	100
Period of publication		
1990–1997	7	4.4
1998–2005	58	36.7
2006–2013	93	58.9
Region		
North America	54	34.2
Europe	91	57.6
Remaining countries^b^	13	8.2
Indicator of socioeconomic position		
Family income	10	6.3
Mother’s and/or father’s education	45	28.5
Mother’s and/or father’s occupation	27	17.1
Various indicators	76	48.1
Method for collecting anthropometric data		
Measured	124	78.5
Reported	32	20.3
Both	1	0.6
No data	1	0.6
Criterion for defining weight status		
IOTF^a^ cut-off pointy	58	36.7
CDC cut-off points	32	20.3
WHO cut-off pints	4	2.5
Other/s criteria/s	64	40.5

^a^IOTF: International Obesity Task Force

^b^Remaining countries included Australia, Israel and Korea

In terms of type of analysis, 68 studies solely performed bivariable analyses, 66 performed bivariable and multivariable analyses, and 24 solely performed multivariable analyses. In all, the papers reviewed provided 134 bivariable and 90 multivariable analyses. Diverse multivariable models were used, in terms both of the number (from two to more than 10) and types of variables of adjustment contained. These variables mainly referred to study participants’ ethnicity or birth weight, various behaviours (breastfeeding, physical activity, sedentarism, consumption of some type of food), parents’ BMI, mother’s characteristics (smoking, age, civil status, depressive symptoms linked to pregnancy) and characteristics of the area of residence (facilities, rurality).

The bivariable analyses showed the presence of an inverse relationship between family SEP and weight status in 60.4 % of cases, an absence of relationship in 18.7 % of cases and a relationship that varied when according to another variable (generally age, sex, ethnicity) in 20.9 % of cases. The multivariable analyses showed an inverse relationship in 51.1 %, an absence of relationship in 20.7 %, a changing relationship according to the breakdown variable in 27.8 %, and a positive relationship in 1.1 % of cases. In some papers, the magnitude of the inverse relationship between SEP and the weight-status indicator was higher for girls than for boys [40, 60, 122, 124, 173]. Similarly, there were 17 studies in which the magnitude of the inverse relationship was greater with obesity than with overweight [61, 64, 87–91, 97, 99, 101, 102, 120, 121, 135, 149, 150, 159] and was likewise greater with severe overweight than with overweight [171] and with morbid obesity than with obesity [47] among the four studies that examined both measures of overweight or obesity [47, 153, 154, 171]. Among the studies in which the relationship varied depending on another variable, mention should be made of some that displayed an inverse relationship in girls but no relationship in boys [30–32, 44, 85, 137, 147, 154], others that displayed an inverse relationship in boys and no relationship in girls [40, 125, 174], and still others that displayed an inverse relationship in white but not in African American participants [30, 31, 41, 50, 57, 65].

Table 3 shows the results for the type of relationship found between family SEP and childhood-adolescent weight status, according to the studies’ period of publication and region of origin. Until 1997 a consistent inverse relationship between socioeconomic status and childhood obesity was not observed. On the basis of the bivariable analyses, the percentage of papers reporting an inverse relationship was 0 % in the period 1990–1997, 62.3 % in the period 1998–2005, and 64.0 % in the period 2006–2013, while the corresponding percentages in the case of the multivariable analyses were 0, 53.6 and 54.4 %, respectively. When viewed by region of origin, the bivariable analyses showed around 60.0 % of papers reported an inverse relationship in all regions; in the multivariable analyses, these percentages were 58.8, 51.1 and 22.2 % in North America, Europe and other regions, respectively.

**Table 3:** Type of relationship observed in the papers according to period of publication and region of origin

Type of relationship	Bivariate analysis (n: 134)		Multivariate analysis (n: 90)	
	n	%	n	%
PERIOD OF PUBLICATION				
1990–1997	6	100.0	5	100.0
Inverse relationship	0	0.0	0	0.0
Absence of relationship	2	33.3	2	40.0
Varies depending on another variable	4	66.7	3	60.0
1998–2005	53	100.0	28	100.0
Inverse relationship	33	62.3	15	53.6
Absence of relationship	10	18.9	5	17.9
Varies depending on another variable	10	18.9	7	25.0
Direct relationship	0	0.0	1	3.6
2006–2013	75	100.0	57	100.0
Inverse relationship	48	64.0	31	54.4
Absence of relationship	13	17.3	11	19.3
Varies depending on another variable	14	18.7	15	26.3
REGION				
North America	44	100.0	34	100.0
Inverse relationship	27	61.4	20	58.8
Absence of relationship	4	9.1	5	14.7
Varies depending on another variable	13	29.5	9	26.5
Europe	77	100.0	47	100.0
Inverse relationship	46	59.7	24	51.1
Absence of relationship	18	23.4	9	19.1
Varies depending on another variable	13	16.9	13	27.7
Direct relationship	0	0.0	1	2.1
Other^a^	13	100.0	9	100.0
Inverse relationship	8	61.5	2	22.2
Absence of relationship	3	23.1	4	44.4
Varies depending on another variable	2	15.4	3	33.3

^a^Remaining countries

One of the main findings in this review is the heterogeneity of age groups analyzed in the included studies. Therefore it is not possible to show the relationship between socioeconomic position and childhood obesity -and / or the magnitude in specific age groups. The exception is the few studies in children under five years, since in some of them the multivariate analysis showed no relationship between maternal education with childhood obesity [114, 132, 139].

Table 4 shows the results for the type of relationship found between family SEP and weight status, according to the SEP indicator and weight-status definition used. The number of analyses carried out with income was small. In the bivariable analyses an inverse relationship was observed in 37.5 % of studies that used family income, in 65.8 % of studies that used the father’s and/or mother’s education, and in 41.7 % of studies that used the father’s and/or mother’s occupation. In the multivariable analyses, the corresponding percentages were 75.0, 62.5 and 41.7 %, respectively. In general, when the education of the mother and father’s education were analyzed, the magnitude of the association was stronger with the education of the mother. For example, in two studies [97, 104] the magnitude of the odds ratios of obesity in the category with lower levels of education with respect to the category with the highest educational level was, respectively, 5.26 and 4.38 with the mother’s education and 4.58 and 2.22 with the father’s education. With some exception, the higher magnitude of the association was observed with income in those studies that analyzed the family income and other measures of income socioeconomic status [19, 33, 57, 58, 61, 62, 122, 131, 136]. Specifically, in these studies, the odds ratio for overweight and obesity in the category of lower family income with respect to the category of higher family incomes ranged between 1.52 [19] and 2.91 [58].

**Table 4:** Type of relationship observed in papers according to socioeconomic position indicator, outcome’s values and criterion used for defining weight status

Type of relationship	Bivariate analysis (n: 134)		Multivariate analysis (n: 90)	
	n	%	n	%
INDICATOR OF SOCIOECONOMIC POSITION				
Family income	8	100.0	4	100.0
Inverse relationship	3	37.5	3	75.0
Absence of relationship	2	25.0	1	25.0
Varies depending on another variable	3	37.5	0	0.0
Mother’s and/or father’s education	38	100.0	24	100.0
Inverse relationship	25	65.8	15	62.5
Absence of relationship	7	18.4	4	16.7
Varies depending on another variable	6	15.8	5	20.8
Mother’s and/or father’s occupation	24	100.0	12	100.0
Inverse relationship	10	41.7	5	41.7
Absence of relationship	9	37.5	5	41.7
Varies depending on another variable	5	20.8	2	16.7
Various indicators^a^	64	100.0	50	100.0
Inverse relationship	43	67.2	23	46.0
Absence of relationship	7	10.9	8	16.0
Varies depending on another variable	14	21.9	18	36.0
Direct	0	0.0	1	2.0
METHOD FOR COLLECTING ANTHROPOMETRIC DATA				
Measured	109	100.0	64	100.0
Inverse relationship	59	54.1	29	45.3
Absence of relationship	24	22.0	13	20.3
Varies depending on another variable	26	23.9	21	32.8
Direct	0	0.0	1	1.6
Reported	23	100.0	24	100.0
Inverse relationship	20	87.0	16	66.7
Absence of relationship	1	4.3	4	16.7
Varies depending on another variable	2	8.7	4	16.7
CRITERION FOR ESTABLISHING WEIGHT STATUS				
IOTF^b^ cut-off points only	50	100.0	36	100.0
Inverse relationship	33	66.0	16	44.4
Absence of relationship	11	22.0	8	22.2
Varies depending on another variable	6	12.0	11	30.6
Direct relationship	0	0.0	1	2.8
Other/s criteria/s	84	100.0	54	100.0
Inverse relationship	48	57.1	30	55.6
Absence of relationship	14	16.7	10	18.5
Varies depending on another variable	22	26.2	14	25.9

^a^Two or three of the above indicators of socioeconomic position were used

^b^IOTF: International Obesity Task Force

When measurements of weight and height were conducted by researchers an inverse relationship was observed in 54.1 % of studies. However, when weight status was derived from self-reported height and weight, an inverse relationship was observed in 87.0 % of studies. In studies in which overweight and obesity were analyzed separately, the magnitude of the association was higher with obesity. For example, the odds ratios in the category with lower education with respect the category with higher parental education in different studies were 1.89 and 2.08 [102], 1.49 and 1.92 [99], 1.43 and 2.10 [86], 1.17 and 2.10 [89], 1.57 and 4.39 [97], 2.16 and 4.10 [149], and 1.42 and 2.82 [150] for overweight and obesity, respectively. One study estimated the magnitude of the association with weight and height measured by researches and weight and height reported by the research subjects [121]. The odds ratio in the children of manual workers with regard to the children of professionals and managers was 1.50 for overweight and 2.67 for obesity when weight and height were measured and 1.36 and 3.67, respectively, when weight and height were self-reported.

In the studies that used the IOFT cut- off points to define weight status, an inverse relationship was found in 66.0 % of the bivariable analyses and in 44.4 % of the multivariable analyses, while in studies that used other criteria to define weight status, an inverse relationship was found in 57.1 and 55.6 % of the bivariable and multivariable analyses, respectively.

## Discussion

This review covered 158 studies undertaken in high income countries and reported from 1990 through 2013. More than half were published in the last 8 years. A total of 60.4 % of papers that performed bivariable analyses observed an inverse relationship between weight status and family SEP, i.e. the measure of weight status registered the highest and lowest magnitudes in participants of low and high SEP, respectively. In contrast, no positive relationship was observed by the papers that performed a bivariable analysis. In the case of papers that conducted multivariable analyses, however, inverse and positive relationships were observed in 51.1 and 1.1 % of cases, respectively.

### Trend in the association between socioeconomic position and weigh status

On the basis of these results and the findings reported by the two earlier reviews published in 1989 and 2008 [10, 11], there is evidence of a change in the pattern of the relationship between SEP and childhood-adolescent weight status in developed countries. Specifically, there has been an increase in the proportion of studies reporting an inverse relationship because in the two earlier reviews, the percentage of papers that observed an inverse relationship stood at around 40 %. Sobal [10] observed a positive relationship in a quarter of the papers included. Accordingly, the presence of positive relationship in only one analysis in the studies included in Shrewsbury and Wardle’s review [11] and in 1.1 % of the multivariable analyses included in our current review, would support the almost disappearance of this relationship.

The increase in the proportion of studies registering an inverse relationship is highlighted when the results are observed by period: while none of the bivariable analyses published in the period 1990–1997 showed an inverse relationship, the number rose to 62.3 % in analyses published in the period 1998–2005 and to 64.0 % in analyses published in the period 2006–2013. More than half of the bivariable analyses were published in the period 2006–2013, which may explain the higher percentage of studies reporting an inverse relationship in our review as compared with the two earlier reviews.

The increase of studies showing inverse relationship between SEP and child obesity reflects increased socio-economic differences in the prevalence of overweight and obesity among adolescents [176]. We can not rule out that this increase may be due to increased economic inequality, since in the last thirty years there was an increase in income inequality in most rich countries [177]. However, it has also been observed an increase in socioeconomic differences in childhood obesity in countries where economic inequality has remained stable. Therefore we should not rule out the influence of other factors, such as the built environment related to physical activity and diet, on the observed results. For example, the availability of supermarkets and stores selling healthy foods has been reported to be lower in areas with lower income than in higher income areas [178], and as result the price of such foods is higher [179]. Likewise, it has been shown that there are fewer sports facilities in lower- than in higher-income areas, so that the probability of engaging in physical activity is also lower [178].

The implications of these findings are very important for health policy and for future research in this area. Although many political interventions warn of the importance of physical activity and healthy diet to prevent childhood obesity, the growing social disparities in child obesity observed in this review reveals the difficulties to reach and communicate health messages to families from lower socioeconomic groups. Some authors have suggested that higher socioeconomic groups tend to follow recommendations for health behaviours and respond more actively to health-related media messages than lower socioeconomic groups [79]. It is also possible that the built environment may create difficulties for the lower socioeconomic groups to adopt healthy behaviours. Future research in this area should be directed to identify why the lower socioeconomic groups do not follow healthy behaviours and evaluate interventions that can be implemented in order to reduce the prevalence of obesity in lower socioeconomic groups.

### Association varies depending on the measurement of outcome and exposure

The relationship between SEP and adiposity differs according to measured or reported weight status. In most of the bivariable analyses based on reported weight status an inverse relationship was found, while this inverse relationship was only found in the half of the bivariable analyses based on measured weight status. The criterion chosen for establishing the weight status cut-off point also influence the type of relationship found, since a higher percentage of performed bivariable analyses found an inverse relationship when internationally accepted cut-off points were used. The same percentage of an inverse relationship was reported in bivariable analyses of studies from North America, Europe and rest of countries. In contrast, the percentage of studies reporting an inverse relationship varied, depending on the SEP indicator used: whereas 65.8 % of studies that performed bivariable analyses using one of the parents’ educational level displayed an inverse relationship, this percentage fell to 41.7 % when the occupation of one of the parents was used to 37.5 % when family household income was used. This finding is of great relevance when it comes to intervention strategies because it may possibly be factors related with parents’ education rather than those related with occupation or household income that are accountable for the inverse relationship between SEP and childhood-adolescent weight status. An unexpected finding with the use of income was the difference in the percentage of inverse relationship in the case of bivariable or multivariable analyses: 37.5 % versus 75.0 %. Probably this inconsistency is due to the number of bivariate and multivariate analysis performed only with family income is very low, 8 and 4 respectively. The increase in the number of studies will confirm or refute this finding.

Shrewsbury and Wardle’s review [11] also found a high percentage of inverse relationship between parents’ education and childhood-adolescent weight status (specifically, in 75 % of studies that used this SEP indicator). The mechanisms whereby the different family SEP indicators influence children’s and adolescents’ weight status are probably different. For instance, parents’ education is associated - to a larger extent than is either occupation or income - with a series of healthy lifestyles that influence children’s and adolescents’ weight status. In the adult population, a strong relationship is observed between education and the prevalence of healthy lifestyles [180–187]. Similarly, a number of studies have pointed that in homes where the parents have a higher educational level, the children are far more likely to follow a healthy diet and be more physically active [188–192]. Something that, at least in part, would be accounted by the influence of the parents’ educational level on attitudes to health and by the exemplary nature of their lifestyles [193–196].

### The importance of other variables in the association

Close on 1 of every 5 papers analyzed shows that the presence and/or type of relationship varies according to other variables, generally age, sex, race and year of study. In any given study, an inverse relationship was seen to appear in the latter years [79, 84, 118] or in the oldest children [94, 105, 113]. In other cases, its magnitude increases with age [25, 116, 153] or is greater in girls than in boys [17, 60, 124, 173] and sometimes this inverse relationship is observed in girls but is absent in boys [30, 32, 44, 72, 78, 83, 137, 147, 153, 154, 173] or is observed only in white participants [18, 30–32, 41, 50, 57, 65]. Similarly, the few positive relationships described affect social minorities [18, 39, 50, 103], i.e. immigrants in Germany, and African Americans and Mexicans in the USA.

In most of the studies, it is difficult to know why the researchers chose to disaggregate the data by social minorities. Perhaps this is due to the acquisition of patterns of risk behaviors. Such habits often first appear among the socioeconomically most privileged and/or indigenous population groups before subsequently appearing among the underprivileged and/or immigrant groups. This might account for the fact that, in developed countries, the inverse relationship between SEP and childhood-adolescent weight status is gradually growing and the positive relationship (peculiar to developing countries) is disappearing. It might also explain why the positive relationship in the case of developed countries is now essentially limited to minorities (certain ethnic groups and the immigrant population). Some authors noted that consumption of calorie-dense foods have increased in developing nations [197]. And it has been observed declines in habitual physical activity, and increases in sedentary behaviour. Traditional practices such as walking long distances, and habitual physical labour have been replaced by motorized transport, and sedentary activities, particularly in urban settings. These factors are now leading to increases in the occurrence of overweight/obesity in developing countries.

Like Shrewsbury and Wardle [11], our review also shows that the inverse relationship displays a greater magnitude with the most severe forms of excess weight (i.e. obesity vs. overweight, or morbid obesity vs. obesity) [47, 61, 64, 87, 88, 90, 91, 97, 99, 101, 102, 135, 159, 171]. This may be due to a mathematical artifact. As a rule, the less frequent the health problem, the greater the magnitude of the relative differences in health among different population groups [196, 198]. The rarer an outcome, the greater tends to be the relative socioeconomic difference in experiencing it. For example, a similar absolute difference in the prevalence of overweight and obesity in two groups - 30 % vs 25 % overweight, and 10 % versus 5 % in obesity- is a relative difference of 1.2 in the first case and 2.0 in the second.

### Strengths and limitations

One aspect to be considered in this review - and indeed one which was also observed by Shrewsbury and Wardle [11] - is that most of the papers reviewed did not designate the relationship between SEP and childhood-adolescent weight status as their main study objective. This was either set a secondary goal, or the authors of the review deduced it from the results reported in the papers. This fact may have led to an underestimate of the number of papers with a statistically significant relationship, since the SEP measures in these cases do not tend to be defined in great detail. Furthermore, the bibliographic review conducted by Shrewsbury and Wardle [11] only used the PubMed database to search for papers. A strength of our review is its comprehensiveness, since the search was made in a number of databases. A further strength is that over half of the papers reviewed were published from 2006 through 2013, a period not covered by the earlier review.

As against an earlier review which only included objective measurements of weight status, ours also included measurements of weight status based on data reported by the study participants themselves, or by their parents or minders. Some authors have stated that reported weight-status data are reasonably valid for classifying children and adolescents as obese or non-obese in epidemiologic studies [199, 200]. Even so, errors of measurement and, by extension, an underestimation or an overestimation of the association cannot be excluded in such studies, if this information bias is different with respect to variables that reflected family SEP. Our findings suggest that a differential bias exists with respect to the SEP when the weight status is reported as almost 90 % of the bivariable analyses found inverse relationship. Publication bias should therefore not be ruled out either. The influence of this bias was probably greater in this than in previous reviews, since our review included a time period during which there was considerable scientific consensus about the inverse relationship between SEP and childhood-adolescent obesity, thereby entailing greater difficulty in publishing results contrary to those expected.

Moreover, one should not lose sight of the limitations attributable to the restrictions imposed in the search, such as language and setting. Studies with languages other than English and Spanish have not been included in this review and have not been included studies from low-income countries. And one may wonder if the search terms in different databases may not have been sufficiently comprehensive. For example, in the PubMed search the term “education” was not included. Likewise, the search in other databases did not include terms like adolescent obesity or overweight or synonyms of social class.

According Petticrew and Roberts [12] one can run a simple check on the effectiveness of a search strategy by listing the key studies that one would expect to identify. These studies can be identified from existing literature reviews. In this sense, this search has found all studies that appear in the review of Shrewsbury and Wardle [11], whose search terms included, among others, education. Likewise, we verified that key articles from our personal database about socioeconomic position and obesity in adolescent appeared among articles that actually retrieved. Our search strategy was not very specific -proportion of retrieved studies that were relevant-, but the search terms and the wide range of databases used probably have enabled high levels of sensitivity to retrieve relevant articles.

In Europe the findings show one or two studies from some countries, such as the Czech Republic, Denmark or Switzerland. Nevertheless, inspection of the results in the countries where several studies have been carried out reveals that in most the observed relationship follows the general pattern, although the social and economic structure of the countries differs between them. However, it should be mentioned Greece, since majority of the studies from that country do not show relationship between SEP and childhood obesity, whereas in southern European countries like Italy or Spain, the observed relationship is mostly inverse.

## Conclusion

The results of this review would seem to confirm the change in pattern which the two previous reviews had already hinted at, and points to the practical disappearance of the positive relationship between SEP and childhood-adolescent obesity in high income countries. Also, this review has shown that the SEP indicator that yields the highest proportion of inverse relationships is parents’ education and that analyses based on the information of height and weight reported by parents obtain higher proportion of inverse relationships that analyses based on objective measurements.

## Additional file

Additional file 1: Table S1. Studies that fulfilled the inclusion criteria. (DOC 460 kb)

## Abbreviations

SEP: Socioeconomic position
WOK: Web of knowledge
Embase: Excerpta Medica Database
OECD: Organization for Economic Cooperation and Development
BMI: Body Mass Index
IOTF: International Obesity Task Force.
