# Supplementary material for: Socioeconomic position and childhood-adolescent weight status in rich countries: a systematic review, 1990–2013
Source: BMC Pediatr. 2015 Sep 21;15:129. doi: 10.1186/s12887-015-0443-3 (PMC4578240; doi:10.1186/s12887-015-0443-3)
Supplement: Additional file 1: Table S1. — Studies that fulfilled the inclusion criteria. (DOC 460 kb) [file 12887_2015_443_MOESM1_ESM.doc]

|  | **AUTOR-YEAR PUBLICATION** | **POPULATION (size, age, sex)** | **DATE** | **OUTCOMES**** | **FAMILIAR SEP** | **BIVARIABLE ANALYSIS RESULTS** | **MULTIVARIABLE ANALYSIS RESULTS** |
| --- | --- | --- | --- | --- | --- | --- | --- |
|  | **AUSTRALIA** |  |  |  |  |  |  |
| **1^108^** | **Wang-2002** | 1581/7-15 y/both | 1995-1996 | Measured. Combined overweight and obesity; IOTF cut-off points | Household income | No significant differences were observed between the different quintiles of household income, except in children in the richest quintile (the prevalence was 50% lower than the prevalence in the poorest quintile). The p values for trend were 0.128 in boys and 0.332 in girls. | N/A |
| **2^109^** | **Wake_2003** | 2862/5-13y/both | 1997 | Measured. BMI. | Paternal and maternal educational level | Inverse relation. Literally: "Higher mean child BMI z-scores was associated with lower maternal and paternal education" | No relationship. After adjusting for BMI maternal and paternal, food intake, organized exercise, television viewing and number of children in the family the relationship with maternal and paternal education disappeared”. |
| **3^106^** | **Mamun-2005** | 3795/14 y/both | 1995-1998 | Measured. Overweight or obesity; IOTF cut-off points | Familiar income and maternal educational level | Only an inverse relationship with family income level (p = 0.007) was observed. To facilitate the understanding of the findings the authors of the review have changed the reference category: age-adjusted OR for family income (reference: high) were 1.24 (0.95-1.64) for medium and 1.35 (1.03-1.79) for low. No relation was observed with maternal education. | N/A |
| **4^113^** | **Timperio-2005** | 291/5-6 y/both and 919/10-12 y/both | 2001 | Measured. Overweight and/or obesity; IOTF cut-off points | Maternal educational level | For 5-6 years there is no association between SEP and overweight or obesity (data not shown). Literally: "None of the variables included in unadjusted logistic regression models were associated with being overweight or obese among 5-6-y-old children (data not presented)." To facilitate the understanding of the findings the authors of the review have changed the reference category: for 10-12 years no association between SEP and risk of overweight was observed but there was an inverse relation with the risk of obesity: OR for maternal educational level (reference: high) were 1.80 (1.00-2.80) for medium and 2.00 (1.11-5.00) for low. | No association with risk of overweight and inverse association with risk of obesity. To facilitate the understanding of the findings the authors of the review have changed the reference category. After for sex, SEP area, car ownership, parental perceptions (heavy traffic and road safety) and perception of child (refer that the parents think that there is heavy traffic in the streets) the OR for obesity (reference: high) was 1.80 if medium and 2.00 if low. |
| **5^114^** | **Wake-2007** | 4983/4-5 y/both | 2004 | Measured. Waist circunference and overweight and obesity. Overweight and obesity: two definitions: a) overweight and obesity; IOTF cut-off points and b) classified as at-risk for overweight (BMI≥ 85 and <95th percentile) and overweight (BMI≥95th percentile) using the CDC definitions | Maternal educational level, occupational class and family income | Inverse relationship was observed with the 3 indicators of SEP. Maternal educational level: OR 0.90 (0.82-0.98), meaning that for every unit increase in maternal educational level the OR of overweight and obesity decreases 0.90. Family income: OR 0 94 (0.89-0.98), meaning that for every decrease of $ 10,000, the OR associated with this decrease is 1.06. Occupational class: with respect to higher category the OR for medium and low categories were 1.34 (1.14-1.57) and 1.54 (1.17-2.01), respectively. | Overweight plus obesity: only inverse relationship with occupation class. Adjusted for sex, number of siblings, birth order, family type, state of residence, urban/rural residence, spoken language mainly in household, aboriginal origin, neighborhood SEP the estimates for occupational class (reference: ASCO 1-3) were: if ASCO 4-7, OR: 1.22 (1.02-1.47), if ASCO 8-9 OR: 1.31 (0.96-1.78) and if not working parent, OR: 1.13 (0.80-1.58). For each increase in maternal education level the OR was 0.97 (0.87-1.08) and for each increase in the level of annual income of $10,000 the OR was 0.99 (0.93-1.06). Waist circumference: no association with any indicator of SEP. |
| **6^105^** | **Hesketh-2007** | 1150/6 y/both and 1370/11 y/both | 2001 and 2002-2003 | Measured. BMI (mean z-BMI) and overweight; IOTF cut-off points | Maternal and paternal educational level | Inverse relationship was found in the whole sample. There was a statistically significant inverse relationship of mean BMI z-score with the education of the mother and the father (p-value <0.001 in both cases). Disaggregated by age and sex, this relationship remains statistically significant inverse in young girls with education of the mother (p-value: 0.02) in young children with education of the father (p-value: 0.04), and in older girls with education of the mother (p-value 0.03) and of the father (p-value 0.007) and in older children with studies of the mother (p-value 0.006) and father (p-value 0.001). To facilitate the understanding of the findings the authors of the review have recalculated the ORs. For overweigh the OR for maternal educational level (reference: high) were 1.20 (0.94-1.51) for medium and 1.41 (1.15-1.75) for low. For overweigh the OR for father´s educational level (reference: high) were 1.32 (1.01-1.72) for medium and 1.47 (1.04-2.04) for low. This relationship disappears for smaller children and remains in the older boys and girls. | Inverse relationship was observed. After adjusting for dual-parent family, number of siblings, parental employment status and also by maternal BMI an inverse association was observed between parental educational level and BMI z-score and risk of overweight in overall sample and in older girls. For older boys only an inverse relationship with BMI z-score was observed. |
| **7^107^** | **Sanigorski-2007** | 2184/4-12 y/both | 2003-2004 | Measured. Combined overweight and obesity; IOTF cut-off points | Household income, Maternal and paternal educational level | There was only inverse relation with the educational level of the father. There was no relationship with the educational level of the mother (p-value: 0.14) or household income (p-value: 0.12). Taking university degree as reference the ORs for father's educational level was 1.19 (0.82-1.73) if father completed technical and further education (TECH), 1.20 (0.81-1.78) if he completed high school, and 1.66 (1.21-2.27) if he did not complete high school. | N/A |
| **8^115^** | **Waters-2008** | 2685/4-13 y/both | 2004-2005 | Measured. Combined overweight and obesity; IOTF cut-off points | Maternal and paternal educational level | Inverse relation was observed with education level of the mother and of the father. To facilitate the understanding of the findings the authors of the review have changed the reference category. Taking university degree as reference the ORs for mother's educational level were 1.58 (1.18-2.16) if she completed 11/12 grade or technical and further education (TECH), and 2.00 (1.59-2.56) if she completed less than or equal to 10 grade. The ORs for father's educational level were 1.83 (1.37-2.43) and 1.85 (1.22-2.78), respectively. Results were also disaggregated by region of birth of mother. When ORs was calculated from the prevalence of overweight plus obesity the ORs for mother's educational level was 1.54 and 1.79 if mothers born in Australia, 1.25 and 1.43 if mothers born in North Africa or the Middle East, and 1.66 and 2.02 if mother born in Europe. | Only inverse relationship was observed with maternal educational level. After adjusting for mothers’ region of birth, possession of a private health insurance policy, neighborhood SEP (index SEIFA), food security, parental weight, age, sex, height and clustering by school, the OR for maternal educational level (reference: university) were: if ≤10 grade, OR: 1.56 (1.19-2.08), if 11/12 or tech, OR: 1.41 (0,98-2,02). |
| **9^111^** | **Gopinath-2012** | 4094/6,7 y and 12,7 y (mean)/both | 2003-2005 | Measured. Overweight and obesity; IOTF cut-off points | Parental educational level (defined as the highest level of education completed by either parent) | N/A | Relationship varies depending on dependent variable. Among 6-year-old children, after adjusting for age, sex, ethnicity, number of siblings and passive smoking, parental education was inverse significantly associated with the prevalence of overweigh and obesity. The reference category was university education. If parental education was less than university the OR was 1.51 (1.15-1.99) for overweigh and 2.06 (1.23–3.46) for obesity. Among 12-year-old children parental education was inverse significantly associated with the prevalence of overweigh only. Additional adjustment for perinatal factors did not modify the magnitude of the association among 6-year-old children, but eliminated the association among 12-year-old children. |
| **10^112^** | **Taylor-2012** | 641/5-15 y/both | 2008-2009 | Reported. Overweight (including obesity); IOTF cut-off points | Maternal educational level and household income | Relationship varies depending on SEP indicator: an inverse gradient was observed in prevalence of overweight according maternal education and household income, although the relation was only significant with maternal education. | No relationship. After adjusting for several socio-demographic and economic variables of household and several variables related with food consumption and physical activity, the relation between maternal education and overweight disappeared. |
| **11^110^** | **Jansen-2013** | 4983/4-5y and 10-11y/both | 2004-2010 | Measured. BMI. | Composite family SEP indicator (including family income, parental educational and occupational level) | An inverse gradient between household SEP position and BMI in all age groups tested was observed. | N/A |
|  | **BELGIUM** |  |  |  |  |  |  |
| **12^152^** | **De Spiegelaere-1998** | 675/3 and 5 y/both | from 1986-90 until 1991-95 | Measured. Excess weight was defined by a BMI > 95th centile of the distribution for age and sex | Parents’ professions and the status of their activity | No association was found either at 3 and 5 years of age. | N/A |
| **13^154^** | **De Spiegelaere-1998** | 2607/12 y/both | 1988-1991 | Measured. Obesity and severe obesity were defined as a BMI of more than 120% and 140% respectively according to the ratio of BMI and the median reference for age and sex of Rolland-Cachera et al | Parents’ professions and the status of their activity | Inverse relationship between SEP and risk of obesity was observed only for girls. The p-values for obesity and severe obesity in girls were 0.0018 and 0.003, respectively. The p-values for obesity and severe obesity in boys were 0.869 and 0.507, respectively. To facilitate the understanding of the findings the authors of the review have recalculated the ORs for girls from the prevalences presented in table 1a: Taking class 1 as reference the ORs for obesity in classes 2,3,4, and 5 were 1.63, 1.83, 1.83 and 2.15, respectively, and the ORs for severe obesity in the same classes were 2.57, 3.68, 2.57 and 5.44, respectively. | N/A |
| **14^153^** | **De Spiegelaere-1998** | 2607/12 and 15 y/both | 1991-1994 | Measured. Obesity if the BMI was >120% of the median for his age and gender. Obesity was considered moderate if the BMI was between 120% and 140% of the median and severe if the BMI was >140% of the median for age and gender. Rolland-Cachera curves. | Parents’ professions and the status of their activity | Literally:" The relative risks of obesity increased between the extreme groups between the ages of 12 and 15. For the girls these relative risks were 1.8 (1.3-2.6) at the age of 12 and 2.1 (1.5-3.1) at the age of 15. For the boys they were 1.0 (0.7-1.5) at the age of 12 and 1.4 (1.0-2.0) at the age of 15”. | N/A |
| **15^155^** | **Moens-2009** | 197/6-14 y/both | No data | Measured. Adjusted BMI (Actual BMI/Percentile 50 of BMI for age and gender *100) and overweight. Overweight if adjusted BMI ≥120%. European BMI values in 0–21 year olds (Fredriks, et al 2000). | Hollingshead Index of Social Position (ISP), which includes parents’ education and occupation | No association was found. | There is no association. |
|  | **CANADA** |  |  |  |  |  |  |
| **16^135^** | **Tremblay-2003** | 7216/7-11 y/both | 1994-1995 | Partially measured and reported (proportion unknown). Overweight and obesity; IOTF cut-off points | A composite index constructed for the National Longitudinal Survey of Children and Youth, comprising parents’ level of education, the prestige of the parents’ occupations, and family income. | Inverse relationship was observed. The authors of the review have calculated OR from the prevalence estimates. Taking as reference the highest category of the indicator of SEP, the ORs for obesity were 1.75 and 2.15 in the middle and in the low categories, respectively. The OR for overweight were 1.39 and 1.81, respectively. | Inverse association was observed. To facilitate the understanding of the findings the authors of the review have changed the reference category. After adjusting for gender, age and single parent (reference: the highest category) the ORs for obesity were: if low, OR: 1.88, if middle, OR: 1.72, and the ORs for overweight were: if low, OR: 1.73, if middle, OR: 1.37. Also adjusted for physical activity the ORs for obesity were: if low, OR: 1.60, if middle, OR: 1.67 and ORs for overweight were: if low, OR: 1.55, if middle, OR: 1.32. |
| **17^133^** | **Willms-2003** | 6060*/7-13 y/both | 1996* (only 1996's) | Reported. Overweight (including obesity); IOTF cut-off points | Familiar income and maternal and paternal educational level | Inverse relationship was observed in Figure 1. Literally: "This figure shows the dramatic inverse relationship between the prevalence of overweight and SEP in all areas of the country". | Only statistically significant inverse relationship was observed with paternal educational level. In a logistic regression model that included household income, mother’s studies, father’s studies, province of residence, age, sex and number of siblings, paternal educational level showed an inverse relationship (OR: 0.96; 0.92-0.99) but not household income or maternal education. |
| **18^136^** | **Veugelers-2005** | 4298/10-11 y/both | 2003 | Measured. Overweight and obesity; IOTF cut-off points | Parental educational level and parental income | Inverse relationship was observed between both indicators and the risk of overweight. Parental educational level (reference: "completed graduate university"): if "completed secondary school or less", OR: 1.56 (1.19-2.04), if "completed community college", OR: 1.36 (1.14-1.61). Parental income (reference: income>60,000 $/y): if < 20,000 $/y, OR: 1.61 (1.25-2.08), if 20,000-40,000 $/y, OR: 1.52 (1.15-2), if 40,000-60,000 $/y, OR: 1.34 (1.03-1.73). | Inverse relationship was observed with parental educational level: Reference category: completed graduate university. After adjusting for lunch type, frequency of family dinner, weekly frequency of participation in physical activity, neighborhood income and weekly physical education classes, the ORs were: if completed secondary school or less, OR: 1.37 (1.05-1.79), if completed community college, OR: 1.33 (1.01-1.77). After adjusting for lunch, family supper, weekly physical activity, sex, neighborhood income and weekly frequency of scholar physical education classes, the ORs were: if completed secondary school or less, OR: 1.82 (1.20-2.78), if completed community college, OR: 1.44 (1.11-1.84). |
| **19^131^** | **Dubois-2006** | 1550/ 4,5 y/both | 1998-2002 | Measured. BMI at or above the 95th percentile at 4.5 years. CDC sex- and age specific growth charts. | Familiar income and maternal educational level | Inverse relationship was observed between overweight and family income but not with maternal educational level. Family income before pregnancy (reference: ≥ $ 60,000), p-value: 0.0013: if <$ 20,000, OR: 2.2 (1.3-3.6). Family income at 4.5 years (p-value: 0.0308): if <$ 20,000, OR: 2.0 (1.1-3.6), if $ 20,000-39,999, OR: 1.6 (1-2.7). | Inverse relationship with family income was observed). Adjusted for gestational age, birth weight, number of parents with overweight or obesity at 18 months of life, maternal smoking during pregnancy and monthly weight gain from birth to 5 months of age, the ORs (reference: income ≥ $ 60,000) were: if income < $ 20,000, OR: 2.5 (1.3-4.8), if $ 20-39,999, OR: 1.6 (1-2.7). |
| **20^132^** | **Dubois-2006** | 1549/4,5 y/both | 2002 | Measured. Overweight as BMI at or above the 95th percentile on the US Centers for Disease Control and Prevention growth charts (2000) and Overweight (including obesity) according IOTF cut-off points | Maternal educational level and household annual income | No association was found | Relationship varies according to the criteria used. No significant relationship was found between mother's education or household income with overweight (either with the criterion of overweight of Cole or CDC). Nevertheless, lowest income category (<$ 20,000) showed an odds ratio almost two times higher than the highest (> $ 60,000). After adjusting for maternal education, parents’ obesity, immigrant status, sex and type of breakfast, the OR in the two lowest income category decreased with overweight criteria CDC and almost unchanged with Cole’s. |
| **21^134^** | **Simen-Kapeu-2010** | 6430/no age (5th grade)/both | 2008 (Alberta)/2005-2009 (Nova Scotia) | Measured. Overweight; IOTF cut-off points | Household income and parental educational level | N/A | Relationship varies with the indicator of SEP: after simultaneous adjustment for parental educational attainment and household income only was observed statistically significant inverse relationship with household income. For all children OR for overweight in level 1 versus level 4 of household income was 1.51 (1.16-1.97) in Alberta and 1.52 (1.13-2.04) in Nova Scotia |
|  | **CZECH REPUBLIC** |  |  |  |  |  |  |
| **22^169^** | **Toschke-2002** | 33768/6-14 y/both | 1991 | Measured. Overweight if BMI>90-th percentile and obesity if >97-th percentile based on the examined population sample | Parental educational level | Inverse relationship was observed. Taking as reference the category of ≥ 10 year of parental educational level, the OR in category for <10 year of educational level was 1.37 for overweight and 1.51 for obesity. | Inverse relationship was observed. To facilitate the understanding of the findings the authors of the review have changed the reference category. After adjusting for being breastfed, parental obesity, maternal smoking, birth weight> 4000 g, daily watching TV> 1 hour, sport outside school and siblings, the OR were: if <10 years, OR for overweight: 1.14 and OR for obesity: 1.23 |
|  | **DENMARK** |  |  |  |  |  |  |
| **23^164^** | **Morgen-2010* (baseline only)** | 3458/14-16 y/both | 1996-1997 | Measured. Overweight (including obesity); IOTF cut-off points | Parental occupational status | N/A | Relationship varies depending on sex. After adjusting for school physician and school cluster effect the ORs (reference: white collar group 1) for girls were: if unemployed, OR 1.88 (1.11-3.21), if non-skilled manual worker, OR: 3.28 (2.10-5.11), if under education, OR: 2.36 (1.07-5.18), if skilled manual worker, OR: 1.41 (0.87- 2.28), if white-necklace group 3, OR: 1.95 (1.30-2.90), if white-collar group 2, OR: 1.51 (1.02-2.24), if self-employed, OR: 3, 63 (2.38- 5.51). In boys there is no relationship |
| **24^165^** | **Rasmussen-2012* (only 15 and 19 y)** | 561/15 y and 19y/both | 1990 and 1994 | Reported. Overweight (including obesity); IOTF cut-off points | Parental occupation level | Literally: "The prevalence of overweight in high- and low socioeconomic position was 3.9 and 4.2% at the age of 15 years, 6.7 and 13.0% at the age of 19 years". | N/A |
|  | **FINLAND** |  |  |  |  |  |  |
| **25^161^** | **Mikkilä-2003** | 60252/14-16 y/both | 1996-1997 | Reported. Obesity if Relative Weight (RW: was expressed as a percentage of the mean weight for sex and height within the sample) >120%. | Parental educational level | N/A | Inverse relationship was observed. After adjusting for school performance, physical exercise, alcohol use, school lunch, dinners at home, fast food consumption the ORs (reference: both have a high school degree) for girls were: if parents without a high school degree, OR: 1.61 (1.37-1.85), if only father has a high school degree, OR: 1.00 (0.81-1.24), if only mother has a high school degree, OR: 1.32 (1.15-1.52). The ORs for boys were: if parents without a high school degree, OR: 1.79 (1.54 -2.08), if only father has a high school degree, OR: 1.43 (1.25-1.71), if mother only has a high school degree, OR: 1.45 (1.27-1.64). |
| **26^160^** | **Kautiainen-2009** | 6503/12,6-18,6 y (mean)/both | 2005* (only 2005's) | Reported. Overweight (including obesity); IOTF cut-off points | Father’s/guardian’s occupational status, father’s/guardian’s education level and mother’s education level | Inverse relationship was observed for boys and girls. Crude ORs have been calculated by the authors of review from prevalence estimates. BOYS: father's occupation level (reference: upper): if low, OR: 1.08, if blue, OR: 1.57, if agriculture, OR: 2.23. Father's educational level (reference: tertiary): if secondary, OR: 1.52, if basic, OR: 1.83. Mother's educational level: if secondary, OR: 1.49, if basic, OR: 1.37. GIRLS: father's occupation level: if low, OR: 1.43, if blue, OR: 1.91, if agriculture, OR: 2.20. Father's educational level: if secondary, OR: 1.22, if basic, OR: 1.76. Mother's educational level: if secondary, OR: 1.12, if basic, OR: 1.81 | N/A |
|  | **FRANCE** |  |  |  |  |  |  |
| **27^122^** | **Klein-Platat-2003** | 3436/12,1 y (mean)/both | 2001 | Measured. Overweight (including obesity); IOTF cut-off points. | Familiar income and parental educational level | Inverse association for both sexes (Table 3). Crude ORs have been calculated by the authors of review from prevalence estimates of overweigh. GIRLS: (reference: high): Family income: OR: 2.26 (none), 2.02 (medium) (p-value <0.001); Parental educational level: OR: 1.92 (low), 1.55 (medium) (p-value <0.0001); Mother's educational level: OR: 2 (low), 1.89 (medium) (p-value: 0.0001); Father's educational level: OR: 1.83 (low), 1.37 (medium) (p-value: 0.001). BOYS: Family income: OR: 1.71 (low), 1.36 (medium) (p-value: 0.01); Parental educational level: OR: 1.61 (low), 1.28 (medium) (p-value: 0.01); Mother's educational level: OR: 1.79 (low), 1.31 (medium) (p-value: 0.001) and father's educational level: OR: 1.77 (low), 1.56 (medium) (p-value: 0.01). | There is only inverse association with maternal educational level. Adjusting for fathers’ and mothers’ obesity and sex the ORs (reference: high) were: if low, OR: 1.62 (1.15-2.27), if middle, OR: 1.48 (1.07-2.05). |
| **28^118^** | **Romon-2005** | 1963: 705 (1989) and 1258 (1999)/5-6 y/both | 1989 and 1999 | Measured. Overweight and obesity; IOTF cut-off points. | Father's occupation (in two-parent family) or single-parent's occupation (in single-parent family) | An inverse association was observed in 1999 but not in 1989. In 1999 social class I had the lowest prevalence of overweight and obesity. Literally: "... in 1989, there were no significant differences between social classes, whereas in 1999 there was a clear difference between classes, both for overweight (p: 0.0005) and obesity (p: 0.04)”. | N/A |
| **29^116^** | **Lioret-2007** | 1016/3-14 y /both | 1998-1999 | Reported. Overweight (including obesity); IOTF cut-off points. | Head of household’s occupation | Inverse association was observed. Crude ORs were calculated by the authors of the revision from the data in Figure 1 (reference: high). By age groups: 3-5 y: if middle, OR: 2.42 and if low, OR: 2.36. 6-10 y: if middle, OR: 1.77 and if low, OR: 3.14. 11-14 y: if middle, OR: 3.62 and if low, OR: 9.75. | Inverse relationship was observed. After adjusting for age, sex, physical activity in leisure time, sedentary lifestyle and daily energy intake, the ORs (reference: high social class) were: in group 3-5 y: if middle, OR: 3.25 (1.50-7.25) and in group 6-14 y: if low, OR: 3.30 (1.43-10.00) and if middle, OR: 1.67 (1.00-2.67). |
| **30^117^** | **Lioret-2009** | 1030/3-14 y/both | 2006-2007 | Measured. Overweight (including obesity); IOTF cut-off points | Head of household’s occupation and educational level | Inversely relation was observed with both indicators of SEP: head of household's occupation (p-value <0.0001) and educational level (p-value <0.0001). | Inverse relationship was observed. After adjusting for age, sex and other two composite SEP indicators (household wealth composite index and SEP composite index) a statistically association was observed with head household’s occupation and educational level. |
| **31^119^** | **Salanave-2009** | 1014/7-9 y/both | 2007 | Measured. Overweight (including obesity); IOTF cut-off points | Parents' occupation and educational level | Inversely relation with parental occupational level was observed. Literally: "Parental occupation was statistically associated with risk of childhood overweight including obesity in both years. In 2000, the prevalence of overweight including obesity was 13.7% for children whose parental occupation was ‘‘managerial or white collar’’, 21.9% for ‘‘self-employed’’, 20.3% for ‘‘employee or manual worker’’ and 23.0% for ‘‘missing data’’ (p= 0.002). In 2007, the prevalence was 10.9%, 17.2%, 18.1% and 21.8%, respectively (p= 0.002)”. | N/A |
| **32^123^** | **Thibault-2010** | 2385/11-18 y/both | 2004-2005 | Measured. Overweight (including obesity) and obesity; IOTF cut-off points | Father's occupation | The relationship varies depending on dependent variable: inverse relationship with overweight (including obesity) and no relation with obesity alone. Crude ORs have been calculated by authors of review from prevalence estimates (reference: high). Overweight (including obesity): if low, OR: 1.90, if medium, OR: 1.58 (p-value <0.01). Only obesity: if low, OR: 1.27, if medium, OR: 1.48 (p-value: 0.58). | Relationship varies depending on dependent variable. Inverse relationship was observed with overweight plus obesity, but no relationship with obesity. After adjusting for sex, age, physical activity, sedentary activity, parental weight, parental physical activity, urban or rural residence the ORs (reference: high) for overweight plus obesity were: if low, OR: 1.78 (1.22-2.60), if medium, OR: 1.44 (1.01-2.05). The ORs for obesity were: if low, OR, 1.07 (0.39-2.89), if medium, OR: 1.40 (0.58- 3.35). |
| **33^121^** | **Chau-2013** | 1559/13 y (mean)/both | 2010 | Measured and reported. Overweight and obesity. French references (Rolland-Cachera) | Paternal occupational level | Inverse relationship. The odds ratio in the children of manual workers with regard to the children of professionals and managers was 1.50 for overweight and 2.67 for obesity when weight and height were measured and 1.36 and 3.67, respectively, when weight and height were self-reported. | Inverse relationship. The magnitude of the association decreased when adjusting for other demographic and socioeconomic characteristics, last-trimester school performance, unhealthy behaviours, physical health, psychological health, social relationships, and living environment and some kind of violence. |
| **34^120^** | **Thibault-2013** | 7667: 4048 (5-7y), 3619 (7-11y)/5-7 y and 7-11 y/both | 2007-09 | Measured. Overweight (including obesity) and obesity; IOTF cut-off points | Parental occupational level | An inverse gradient between parental occupation and overweight and obesity was observed, although magnitude of association was greater in obesity. | Inverse relationship. Adjustment for variables related to eating behavior and physical activity did not substantially change magnitude of association. |
|  | **GERMANY** |  |  |  |  |  |  |
| **35^92^** | **Kromeyer-Hauschild-1999** | 1901/7-14 y/both | 1995* (only 1995's) | Measured. Overweight if BMI>90-th percentile of the Rolland–Cachera curves | Paternal occupation and maternal educational level | N/A | Inverse relationship was observed with fathers’ occupation for boys and girls. The OR (reference: highly qualified) for boys, adjusting for age and birth weight was: if manual worker, OR: 2.37 (1.28-4.38). The OR for girls, adjusting for age, birth weight and size of the apartment was: if manual worker, OR: 2.06 (1.19-3.57). |
| **36^101^** | **Von Kries-1999** | 9357/5-6 y/both | 1997 | Measured. Overweight if BMI>90-th percentile and obesity if >97-th percentile. Regional curves. | Parental educational level | Inverse association was observed with overweight and obesity. Taking as reference 10 or more years of education: if <10 y: overweight, OR: 1.29 (1.12-1.49) and obesity, OR: 1.61 (1.26-2.04). | Inverse relationship was observed. After adjusting for exclusive breastfeeding, maternal smoking during pregnancy, low birth weight, having own bedroom and eat butter 3 or more times per week, The ORs (reference: ≥ 10 y of studies) for overweight was OR: 1.23 (1.05 -1.43) and for obesity: 1.33 (1.02 -1.72). |
| **37^96^** | **Müller-1999** | 1468/5-7 y/both | 1995-1997 | Measured. BMI and overweight. Overweight defined as a Triceps skinfold thickness of > 90-th percentile according to reference data from the Forschungsinstitut für Kinderernährung in Dortmund | Parental educational level | Inverse relationship was observed. Literally: "Mean group BMI and also the prevalence of overweight were influenced by the social status showing highest values in children from a low social status …. Social status was defined by school education level. Low vs. middle: p<0.001, low vs high: p<0.001, middle vs high: n.s." | N/A |
| **38^93^** | **Langnäse-2002** | 1350/5-7 y/both | 1996-1998 | BMI and Triceps skinfold thickness (TSF) measured; percentage-fat mass (FM) calculated; overweight. Overweight (as defined by the 90-th percentile of weight, BMI, TSF and percentage FM). The 90-th percentile of weight, BMI, and percentage FM, derived from the total sample of the Kiel Obesity Prevention Study, obtained between 1996 and 1998. The 90-th percentile of TSF according to references of the Forschungsinstitut für Kinderernährung, Dortmund. | Parental educational level | Inverse relationship was observed. ANOVA p-value for mean values are significant for social class: BMI (p = 0.0001), TSF (p: 0.002) and fat mass (p = 0.0001). | N/A |
| **39^102^** | **Von Kries-2002** | 6483/5-6,99 y/both | 1999-2000 | Measured. Overweight if BMI> 90-th percentile and Obesity if > 97-th percentile. Regional curves. | Parental educational level | Inverse relationship was observed with overweight and obesity. If <10 y schooling compared to 10 or more years: overweight, OR: 1.89 (1.59-2.22) and obesity, OR: 2.08 (1.54-2.78). | Relationship varies depending on the dependent variable. To facilitate the understanding of the findings the authors of the review have changed the reference category. After adjusting for maternal smoking during pregnancy, obesity of any parent, birth weight> P90, watch TV or play video games >1 h per day, received some breastfeeding and eating snacks while watching TV, the OR (reference: ≥ 10 y of education) for overweight if < 10 was 1.43 (1.12-1.82) and for obesity 1.47 (0.97-1.22). |
| **40^94^** | **Langnäse-2003** | 1326/5-7 y/both | 1996-1998 | Measured. Overweight if BMI≥ 90-th percentile. German reference data. | Parental educational level | Only there is a statistically significant inverse relationship with the parents' educational level at 5-7 y (p-value <0.05). No relationship at 1 or 2 years old. | N/A |
| **41^88^** | **Bergmann-2003** | 480/6 y/both | 1996 | Measured. Overweight if BMI and Skinfold thickness are ≥90-th and <97-th percentile and Obesity if ≥97-th percentile. BMI: Rolland-Cachera curves. Skinfold thickness: age and gender-specific values by Prader et al served as the standards. | Maternal educational level and Social index of the family (containing educational and occupational level of the parent contributing the highest share to the family income and family income itself) | Inverse relation was observed with both SEP indicator: Social Index of the family (p-value: 0.0005), maternal educational level (p-value: 0.0005). Crude ORs were calculated by authors of review from prevalence estimates (reference: high). OVERWEIGHT: Social Index of the family: if low, OR: 1.29, if middle, OR: 1.49. Maternal educational level: if low, OR: 1.27, if middle, OR: 1.56. OBESITY: Social Index of the family: if low, OR: 2.98, if middle, OR: 1.91. Maternal educational level: if low, OR: 2.71, if middle, OR: 1.44. | Inverse relationship was observed between composite index and overweight and obesity after adjusting for breastfeeding, maternal overweight and maternal smoking during pregnancy. |
| **42^99^** | **Toschke-2003** | 4974/5-6 y/both | 2001-2002 | Measured. Overweight and obesity; IOTF cut-off points | Parental educational level | Inverse association was observed. Reference: ≥ 10 y: if <10 y, for overweight OR: 1.49 (1.25-1.82) and for obesity OR: 1.92 (1.39-2.70). | N/A |
| **43^90^** | **Frye-2003** | 6650/5-7 y and 8-10 y and 11-14 y/both | 1992-1993; 1995-1996; 1998-1999 | Measured. Overweight and obesity; IOTF cut-off points | Parental educational level | Inverse association was observed: clearer for obesity than for overweight. Reference: university degree: OVERWEIGHT: if less than 10 grades, OR: 1.43 (1.11-2), if 10 grades, OR: 1.43 (1.14-1.86), if 12 grades, OR: 1.43 (1-1.86). OBESITY: if less than 10 grades, OR: 2.50 (1.25-5), if 10 grades, OR: 2.75 (1.75-4.25), if 12 grades, OR: 1.75 (1-3). In overweight there is a difference between those with college and the rest. The lower prevalence of overweight was observed in children of parents with college while in other educational categories the prevalence of overweight was about 40% higher | N/A |
| **44^89^** | **Danielzik-2004** | 2631/5-7 y/both | 1996-2001 | Measured. Overweight if BMI ≥90-97-th and Obesity if >97-th percentile. German BMI percentiles. | Parental educational level | Inverse relationship was observed for overweight y obesity. Crude ORs calculated (reference: high). Overweight: if low, OR: 1.17 and if middle, OR: 1.33. Obesity: if low, OR: 2.10 and if middle, OR 0.92. Literally: "There was an inverse gradient in SEP: when compared with high SEP, children from low SEP families were more frequently overweight." | Inverse relationship was observed with overweight and obesity in boys and with overweight in girls. After adjusting for single parenthood, parental obesity, parental smoking and parental overweight, the OR for overweight in boys in low versus high SEP was 9.8 (1.8-53.1) and after adjusting for parental smoking, low birth weight, obese parents and single parenthood, the OR for obesity in boys in low versus high SEP was OR 9.3 (1.6-51.9). After adjusting for single parenthood, parental obesity, parental smoking and parental overweight, the OR for overweight in girls in low versus high SEP was 2.1 (1.1-4.2). |
| **45^103^** | **Will-2005** | 525/6-7 y/both | 2002 | Measured. Overweight; IOTF cut-off points | A social class index was constructed using four indicators (primary qualification, professional education, employment status and size of household). | Relationship varies depending on origin of the minor: direct in migrants and inverse in Germans. Crude ORs is estimated by authors of the review from prevalence estimates (reference: high). Migrants: if low, OR: 0.57, if medium, OR: 0.31. Gemans: if low, OR: 2.76, if medium, OR: 0.73. | Multivariate analysis performed only on immigrants: a direct relationship was observed. The OR (reference: high) adjusted for sex, origin and length of stay in Germany was: if low, OR: 0.61 (0.31-1.20) and if medium, OR: 0.35 (0.20-0.60). |
| **46^104^** | **Lamerz-2005** | 1979/5-7 y/both | 2001-2002 | Measured. Obesity if BMI≥ 90-th percentile. Regional curves. | Maternal and paternal educational level and a social strata index (containing: parental education, living space in square meter per person living in the household and single parenthood). | Inverse relationship was observed: Maternal educational level (reference: 13 y): if no education, OR: 5.26 (3.11-8.92) and if 9y, OR: 2.38 (1.58- 3.58). Paternal educational level: if no education, OR: 4.58 (2.57-8.16), if 9 y, OR: 2.27 (1.52-3.38) and if 10-12 y, OR: 1.63 (1.04-2.56). Social strata index (reference: 4 to <6 points): lowest social level (10-15 points): OR: 3.29 (1.92-5.63). | Relationship varies with the indicator of SEP: inverse relationship was observed with maternal level education and no association with paternal education. For maternal educational level (reference: 13 y) the OR adjusted for sex, maternal BMI, paternal BMI, maternal and paternal employment (full time, part time and none), habitable square meters per person and single parenthood was 2.86 (1.36-6.03) if no degree. |
| **47^95^** | **Mikolajczyk-2008** | 5650/11-17 y/both | 2001-2002 | Reported. Overweight if BMI ≥90-th percentile. Age- and gender-specific BMI cut-off points for the German population. | Parental occupation | Inverse relationship was observed for boys and girls. Boys (p-value <0.05) and girls (p-value <0.05). Crude OR are calculated by authors of the review from prevalence estimates (reference: high): Boys: if middle, OR: 1.32 and if low, OR: 2.02. Girls: if middle, OR: 0.89 and if low, OR: 1.47. | No relationship was observed. After adjusting for sedentary behavior, sweets and sugary drinks, the material welfare of the family, make diet, school, and geographic region of residence, for the overall sample, no relationship between parental occupation and risk of overweight was observed. |
| **48^87^** | **Apfelbacher-2008** | 35434/5-7 y/both | 1991-2000 | Measured. Overweight and obesity; IOTF cut-off points | Parental educational level | Inverse relationship was observed. Overweight (educational level as a continuous variable): for each increased unit, OR: 0.79 (0.75-0.82). Overweight (educational level as a categorical variable and reference: >10 y): if <10 y, OR: 1.58 (1.44-1.75) and if 10 y, OR: 1.28 (1.17-1.39). Obesity (educational level as a continuous variable): OR: 0.68 (0.63-0.73). Obesity (educational level as and as a categorical variable and reference: >10 y): if <10 y, OR: 2, 08 (1.78-2.43) and if 10 y, OR: 1.60 (1.37-1.87). | Inverse relationship was observed. There is an inverse relationship between parental educational level (as continuous variable) and the risk of overweight and obesity. The OR adjusted for sex, age, other than German nationality, study region (East / West), study location (urban / rural), study year, living space> 75 m2, number of persons, single child, attendance of day nursery, smoking in the living place, passive smoking during the first three years of life, smoking during pregnancy, breastfeeding for more than three months, preterm delivery, and birth weight was 0.87 (0.82-0.92) for overweight and 0.80 (0.73-0.89) for obesity |
| **49^97^** | **Nagel-2009** | 1079/6-9 y/both | 2006 | Measured. Overweight and obesity; IOTF cut-off points | Maternal and paternal educational level | Inverse relationship was observed. Overweight: there was a statistically significant inverse relationship with maternal and paternal educational level. Obesity: there was a statistically significant inverse relationship only with maternal educational level. Reference: > 10 y). Overweight: maternal educational level, if 10 or fewer y, OR: 1.57 (1.05-2.33) and paternal educational level, if 10 or fewer y OR: 1.79 (1.22-2.64). Obesity: maternal educational level, if 10 or fewer y, OR: 4.39 (1.34 -14.41) and paternal educational level, if 10 or fewer, OR: 2.22 (0.96-5.11). | N/A |
| **50^91^** | **Kleiser-2009** | 13450/3-17 y/both | 2003-2006 | Measured. Overweight (including obesity) and obesity; IOTF cut-off points | SEP level assesed (parents' income, occupational status and education). | Inverse relationship was observed. Crude OR are calculated by authors of the review from prevalence estimates (reference: high): if low, OR: 2.14 and if medium, OR: 1.50. Obesity: if low, OR: 3.81 and if medium, OR: 1.92. | Inverse relationship was observed. After adjusting for age and sex, the ORs for obesity plus overweight (reference: high) was: if low, 2.12 (1.80-2.40), if medium, 1.47 (1.30-1.70). The ORs for obesity only were: if low, OR: 3.76 (3-4.7), if medium, OR: 1.87 (1.4-2.5). |
| **51^98^** | **Plachta-Danielzik-2010** | 1087/5-11 y/both and 6249/5-16 y/both | 1996-2006 | Measured. Overweight (including obesity); IOTF cut-off points | Parental educational level | N/A | Inverse relationship with overweight in girls was observed. After adjusting for family factors, social factors, birth weight, lifestyle, age and pubertal stage, the OR in low vs high social status was OR: 1.6 (1.2-2.1). |
| **52^100^** | **Beyerlein-2011** | 13223/3-17y/both | 2003-2006 | Measured. BMI: | SEP was classified based on the parents’ professional status, income and educational achievements and assigned to low, middle or high according to the parent with the higher status | N/A | Relationship varies according age. After adjusting for maternal BMI, maternal smoking in pregnancy, low parental socioeconomic status was no associate with BMI-z score in children 3 to 10 years old. However, a positive and significant association was observed for percentiles 70, 80, 90 and 97 of BMI-z score in children 11 to 17 years old. |
|  | **GREECE** |  |  |  |  |  |  |
| **53^142^** | **Panagiotakos-2008** | 700/10-12 y/both | 2005-2006 | Measured. Combined overweight and obesity; IOTF cut-off points | Maternal and paternal educational level | No relationship was found either boys or girls | N/A |
| **54^143^** | **Yannakoulia-2008** | 1138/11,2 y (mean)/both | No information | Measured. Overweight; IOTF cut-off points | Maternal and paternal educational level | No relationship was found | N/A |
| **55^144^** | **Moschonis-2010** | 729/9-13 y/both | 2007 | Measured. Overweight plus obesity; IOTF cut-off points | Paternal and maternal educational level and annual family income | No relationship with the three SEP indicators. In the case of family income the higher prevalence was observed in an intermediate category. | No relationship. Adjusted for parent’s nationality, residence ownership, child’s primary caregiver, popularity score, gender and for the clustering effect of schools, the relation of family income with overweight was not altered. |
| **56^146^** | **Cassimos-2011** | 335/11,02 y (mean)/both | 2008-2009 | Measured. Overweight plus obesity; IOTF cut-off points | Paternal and maternal educational level | No relationship was found | N/A |
| **57^145^** | **Farajian-2012** | 2315/10-12 y/both | 2009 | Measured. Overweight/obesity; IOTF cut-off points | Maternal and paternal educational level, maternal and paternal occupation and family income | Inverse relationship. The odds of being an overweight/obese child were reduced when the mother’s or father’s type of occupation tended to be non-manual and the same effect was also noticed regarding father’s high educational status. | Relatioship depends on gender. After adjusting for child´s age, mother´s age, paternal occupation and education, place of residence, family income and parental obesity status, no relationship was observed between SEP indicators and overweight/obesity in boys. However, among girls, family income and father’s occupation were negatively related. |
|  | **HOLLAND/NETHERLANDS** |  |  |  |  |  |  |
| **58^147^** | **Boot-1997** | 403/4-20 y/both | No information | Calculated on measures. Percentage body fat and fat mass. | Maternal and paternal educational level and parents' occupation | Data not shown: the results are presented in the text. Relationship varies with sex. Boys: no relationship. Girls: statistically significant inverse relationship between parental occupation and paternal educational level with the percentage of body fat (p-value: 0.008 and 0.002, respectively) and fat mass (p-value: 0.003 and 0.0002, respectively). No relation with maternal educational level. | Data are not shown. Results are extracted from text. For girls and adjusting for Tanner stage and physical activity, inverse relation only with paternal educational level (p-value: 0.03 for fat mass and p-value: 0.01 for the percentage body fat). |
| **59^148^** | **Fredriks -2000** | 14500/0-21 y/both | 1996-1997 | Measured. BMI. | Parental educational level | Inverse relationship was observed. Literally: "Figure 3 shows the effects of demographic variables on BMI-SDS. Univariately, mean BMI was significantly related to educational level of the parents (p <0.0001)". | N/A |
| **60^149^** | **Veldhuis-2013** | 5582/5 y/both | 2007-2008 | Measured. Overweight and obesity; IOTF cut-off points | Maternal educational level | Statistically significant inverse relationship with obesity and overweigh. The ORs of low and mid versus high educational level were 1.52 (1.18-1.96) and 2.16 (1.62-2.88) for overweight and 1.44 (0.80-2.61) and 4.10 (2.28-7.35) for obesity, respectively. | Inverse relationship. After adjusting for characteristics of the mother (age, single parenting, employment status, weight status and watching TV) and child (sex, age, consuming breakfast, drinking sweet beverages, playing outside and watching TV) the magnitude of the inverse association decreased. Model 3 (adjusting for maternal age, weight status and watching TV and child consuming breakfasts and watching TV): the ORs of mid and low versus high educational level were 1.36 (1.05-1.77) and 1.81 (1.33-2.46) for overweight and 1.19 (0.64-2.18) and 2.84 (1.52-5.29) for obesity, respectively. |
| **61^151^** | **Van den Berg-2013** | 1965/5-6 y/both | 2008-2009 | Measured. BMI, Fat Mass Index and Lean Mass Index | Maternal educational level | Relationship varies depending on dependent variable: children with low-educated mothers had a higher BMI, and higher fat mass index (FMI) compared to children with high-educated mothers-. | Relationship varies depending on dependent variable. After adjustment for confounders -child’s age, sex, maternal age, maternal smoking, and maternal BMI- there were no educational differences in BMI but children with low-educated mothers had a higher FMI (β 0.28; 95% CI 0.07 - 0.49) compared to children with high-educated mother |
| **62^150^** | **Ruijsbroek-2011** | 3963/3-8 y/both | From 1996-97 until 2004-05 | Reported. Overweight (including obesity) and obesity; IOTF cut-off points | Maternal educational level | Significant inverse relationship. With respect to the group with high maternal education, the odds ratio for overweigh and obesity in the group with low maternal education was 1.42 and 2.82, respectively. | Significant inverse relationship. After adjusting for smoking during pregnancy, smoking in the home, and day-care centre attendance the odds ratio for overweigh decreased to 1.26 and after adjusting for smoking during pregnancy, smoking in the home, breastfeeding and day-care centre attendance the odds ratio for obesity decreased to 2.04. |
|  | **ICELAND** |  |  |  |  |  |  |
| **63^170^** | **Magnusson-2008** | 488/9 y/both | 2003-2004 | Measured. Sum of the four skinfold thickness (tricep, bicep, subscapular and suprailiac) -SKF-, | Maternal and paternal income and educational level | No relationship was observed | N/A |
|  | **IRELAND** |  |  |  |  |  |  |
| **64^167^** | **Griffin-2004** | 199/11 y (mean)/both | 1999-2000 | Measured. Overweight if BMI ≥ 26 kg/m**^2^** | Parental (or guardian's) occupational level | Literally: "Anthropometric measurements did not differ according to socio-economic class (SEC) classification". And "The prevalence of BMI ≥ 26 kg/m^2^ did not differ according to SEC classification". | N/A |
| **65^166^** | **Keane-2012** | 8568/9 y/both | 2007-2008 | Measured. Overweight and obesity; IOTF cut-off points | Household occupational level, household income and maternal educational level | N/A | Relationship varies depending on dependent variable. After adjusting for gender, parent family type, presence of siblings, parent weight status and the three indicators of SEP, it was not observed relation between the three indicators and overweight. Nevertheless, after the adjustment a significant inverse relationship was observed between maternal education and household social class household with obesity. |
|  | **ISRAEL** |  |  |  |  |  |  |
| **66^171^** | **Huerta-2006** | 8623/8-13 y/both | 1997 and 2000 | Measured. BMI (mean BMI), overweight (if BMI > 85-th percentile) and severe overweight (if > 95-th percentile). | Maternal and paternal educational level and an additional composite variable constructed to reflect combined parental education ("Combined Parental Education" ) | Inverse relationship was observed. Mean BMI: an inverse relationship only with father's educational level (p-value: 0.05). Overweight: there was an inverse relationship with the 3 indicators of SEP (p-value <0.001). Severe overweight: there was only an inverse relationship with father's educational level (p-value <0.001) and the combined indicator (p -value 0.01). Overweight: taking some college or more as the reference category for educational level, the OR for high school or less of the father was 1.23 (1.07-1.41) and the OR for high school or less of the mother was 1.23 (1.08-1.40). Overweight: taking both attended college the reference category for combined index the ORs were 1.23 (1.01-1.50) if one attended college and 1.32 (1.12-1.54 if neither attended college. Severe overweight: the OR for high school or less of the father was 1.30 (1.04-1.63) and the OR for high school or less of the mother was 1.21 (0-99-1.50). Severe overweight: the OR if one attended college was 1.40 (1.08-1.82) and the OR if neither attended college was OR: 1.49 (1.09-2.04). | Inverse relationship was observed. After adjusting for number of smoker parents, age, sex, immigrant status, the ORs for overweight (reference: both parents attended college) were: if neither attended college, OR: 1.21 (1.03-1.42), if one attended college, OR: 1.19 (0.98-1.45). And the ORs for severe overweight were: if neither attended college, OR: 1.49 (1.09-2.05), if one attended college, OR: 1.31 (1.04-1.71). |
|  | **ITALY** |  |  |  |  |  |  |
| **67^157^** | **De Vito-1999** | 1357/11-19 y/both | 1997-1998 | Measured. At risk of overweight when BMI ≥85-th percentile and both tricipital and subscapular skinfolds < 90-th percentile. Obesity when BMI was ≥85-th percentile and both tricipital and subscapular skinfolds ≥90-th percentile. WHO standard population. | Maternal and paternal educational level | No relationship was found with either maternal or paternal education. | N/A |
| **68^159^** | **Gnavi-2000** | 1420/10-11 y/both | 1998 | Measured. Overweight if Relative Weight (RW= measured weight/weight at 50-th centile for statural age and gender*100) was between 120 and 139% and obesity if RW ≥140%. Weight at the 50-th centile for statural age and gender of each child was calculated using NCHS growth tables. | Maternal and paternal occupation and educational level | Inverse relationship was observed. ORs are calculated by authors of the review from prevalence estimates. Overweigh. Maternal educational level (reference: degree or high school): if middle, OR was 1.07 and if elementary or low, OR was 1.55. Maternal occupation (reference: upper non manual): if low non manual, OR was 1.12 and if manual, OR was 1.70. Paternal educational level: if middle, OR was 1.25 and if elementary or low was OR: 0.89. Paternal occupation: if low non manual, OR was 1.31 and if manual, OR was 1.68. Obesity. Maternal educational level: if middle, OR was 1.49 and if elementary or low, OR was 2.20. Maternal occupation: if low non manual, OR was 1.18 and if manual, OR was 2.74. Paternal educational level: if middle, OR was 1.39 and if elementary or low, OR was 2.71. Occupation father: if low non manual, OR was 0.83, if manual OR was 3.36. Overweigh and obesity. Maternal educational level: if middle, OR was 1.18 and if elementary or low, OR was 1.72. Maternal occupation: if low non manual OR was 1.14 and if manual OR was 1.96. Paternal educational level: if middle, OR was 1.29 and if elementary or low, OR was 1.34. Occupation father: if low non manual, OR was 1.16 and if manual, OR was 1.72. The p values for trend for overweight and obesity together were 0.015, 0.001, 0.002, 0.001 for: parental educational level, parental occupation, maternal educational level and maternal occupation, respectively. | An inverse association was observed. Prevalence ratio adjusted for maternal age, school district and parental area of birth are estimated. The findings for overweight and mother's education (reference: college) were 1.72 (1.32-2.23) if elementary education or less, PRR: 1.72 (1.32-2.23). The findings for overweight and father's education (reference: college) were: if elementary education or less, 1.34 (1.01-1.78), if medium studies, 1.29 (1.04-1.59). The findings for overweight and mother's occupation (reference: non-manual superior works) were: if manual, 1.96 (1.29 -2.97), if housewives, 1.47 (1-2.17), if unemployed, 2.57 (1.54-4.28). The findings for overweight and father's occupation (reference: non-manual superior works) were: if manual, 1.72 (1.34-2.19), if unemployed, 3 (1.97-4.57). The findings for obesity and mother's education were 1.59 (1.19-2.13) if elementary education or less. The findings for obesity and mother's occupation were: if manual, 1.83 (1.20-2.79), if unemployed, 2.20 (1.31-0.68). The findings for obesity and father's occupation were: if manual, 1.63 (1.27-2.09), if unemployed, 2.63 (1.72-4.03). |
| **69^158^** | **Giampietro-2002** | 869/9,8 y (mean)/both | 1996-1997 | Measured, BMI. | Maternal and paternal educational level | Inverse relationship with father (p-value <0.01) and mother educational level (p-value <0.05) | N/A |
| **70^156^** | **Celi-2003** | 12143/3-17,5 y/both | 1993-2001 | Measured. Overweight and obesity according to two different definitions: (1) the 90-th and the 97-th percentile BMI, respectively, of the French standard of Rolland-Cachera et al (1991) and (2) IOTF cut-off points. | Parental occupation | Inverse relationship with overweight (p-value <0.01) and obesity (p-value <0.01) was observed. Literally: "The association with the socioeconomic status of the families was analysed as a trend according to the values attributed in univariate analysis; a negative trend for overweight (p <0.01) and for obesity (p <0.01) was found, with higher percentage among the lowest socioeconomic levels". | N/A |
|  | **KOREA** |  |  |  |  |  |  |
| **71^172^** | **Lee-2013** | 7879/2-18 y/both | 2007-2010 | Measured. Overweight plus obesity and obesity alone; IOTF cut-off points. | Parental educational level and Household income. | No relationship was observed. | No relationship was observed. The variables included in the model were: age, gender, region, income category and education of adult in a household with children. |
|  | **NORWAY** |  |  |  |  |  |  |
| **72^173^** | **Lien-2007** | 5498/15-16 y/both | 2000-2001 | Reported. BMI and overweight (including obesity); IOTF cut-off points. | Parental income and educational level | Inverse relationship was observed. Male: both the mean value of BMI as to prevalence of overweight / obesity are inversely related to the parents' educational level (p-value <0.001) but not with that of parental income. Women: both the mean value of BMI as to prevalence of overweight / obesity are inversely related to the parents' educational level (p-value <0.001) and income level (p-value: 0.001 and 0.01 respectively). ORs are calculated by authors of the review from prevalence estimates of overweight / obesity. Boys: for parents' educational level (reference: master), if primary, OR was 2.79, if secondary, OR was 1.94 and if bachelor OR was 1.44. Girls: for parents' educational level (reference: master), if primary, OR was 3.08, if secondary, OR was 1.66 and if bachelor, OR was 1.60. For parental income level (reference: 5th quintile), if first quintile, OR was 2.04, if second, OR was 2.23, if third, OR was 1.27 and if fourth, OR was 1.32. | Inverse relationship with parental educational level was observed, but no with parental income after adjusting for (area of residence and family model. |
|  | **PORTUGAL** |  |  |  |  |  |  |
| **73^162^** | **Padez-2009** | 4511/7-9 y/both | 2002-2003 | Measured. Combined overweight and obesity; IOTF cut-off points | Maternal and paternal educational level | There is a statistically significant relationship with maternal and paternal educational level for both boys and girls, but only in girls there is an inverse gradient. | Inverse relationship was observed. To facilitate the understanding of the findings the authors of the review have changed the reference category. After adjusting for father and mother obesity, daily hours of physical activity and TV the OR for paternal educational level (reference: university) were: if primary, 1.41 (1.32-1.54), if secondary, 1.48 (1.41-1.54). The OR maternal educational level were: if primary, 1.25 (1.18-1.33), if secondary, 1.38 (1.31-1.43). |
| **74^163^** | **Nogueira-2013** | 1885/3-10 y/both | No data | Measured. Obesity; IOTF cut-off points | Parental educational level | Inverse relationship. Children from low and medium SEP backgrounds were more likely to be obese than their high SEP peers. | Inverse relationship. Adjusting for the child’s gender, age and the clustering of children in schools did not change the magnitude of the association: the odds ratio for low and medium SEP backgrounds were 1.767 and 1.574, respectively |
|  | **SPAIN** |  |  |  |  |  |  |
| **75^126^** | **Serra-2003** | 3534/2-24 y/both | 1998-2000 | Measured. Overweight plus obesity (if BMI≥85-th percentile) and obesity (if BMI≥97-th percentile). Spanish reference data (Hernández et al, 1998). | Head of household’s occupation | Inverse relationship was observed for obesity (p-value: 0.005) and for overweight and obesity (p-value: 0.002). | N/A |
| **76^125^** | **Moreno-2004** | 2851*(only AVENA study)/13-18,5 y /both | 2000-2002 | Measured. Overweight and obesity; IOTF cut-off points | SEP level assesed (educational level and type of occupation of both parents). | Inverse relationship was observed. In boys there was a statistically significant association with maternal educational and occupational level. In girls there was only a statistically significant association with maternal educational level. Literally: "In male adolescents, the variables that showed significant differences between overweight and non-overweight individuals were age, maternal occupation, maternal employment status, maternal education level, maternal obesity, and paternal obesity [...] In females, the variables that showed significant differences between groups were age, maternal education level, and maternal obesity". | Inverse relationship with maternal studies was observed only for boys. From text: “maternal education and paternal history of obesity were associated with overweight but when were included together in the model only maternal education was significant in boys but not in girls”. |
| **77^127^** | **Vicente-Rodriguez-2008** | 1960/13-18,5 y/both | 2000-2002 | Measured. Combined overweight and obesity; IOTF cut-off points | SEP level assesed (father's educational level and type of occupation) | Inverse relationship was observed. The OR for SEP as a continuous variable was 0.875 (0.774- 0.989). Literally: "Overweight risk [...] and an increase of one level in socioeconomic status reduced the risk by 12.5%." | N/A |
| **78^124^** | **Bibiloni-2010** | 1231/12-17 y/both | 2007-2008 | Measured. Obesity if BMI ≥ 97-th percentile. WHO growth standards. | Parents' occupation and educational level | Inverse relationship with parental educational level was observed. Taking as reference the high educational level, the OR for low educational level was 2.99 (1.46-6.12) in boys and 3.75 (1.60-8.79) in girls. | There was an inverse relationship with parental educational level for boys and girls. After adjusting for age, number of daily hours of sleep and number of daily meals the ORs for parental educational level (reference: high) in boys were: if low, OR: 3.47 (1.58-7.62), if medium, OR: 2.35 (1.04-5.34). In girls the ORs were adjusted for number of daily meals and physical activity level were: if low, 3.29 (1.38-7.89). |
| **79^129^** | **Navalpotro-2012** | 4528/6-15 y/both | 2006-2007 | Reported. Overweight and obesity; IOTF cut-off points | Primary household earner's educational level and occupation | Statistically significant inverse relationship (p <0.001) with both indicators of SEP for both obesity and overweigh. The ORs in low versus high educational level were 1.58 for obesity and 1.59 for overweigh. The ORs in manual versus non-manual occupation were 1.75 for obesity and 1.49 for overweigh | N/A |
| **80^128^** | **Costa-Font-2013** | 13358/2-15y/both | 2003-04 and 2006-07 | Reported. Obesity; IOTF cut-off points | Household income, maternal and paternal educational level. | N/A | Inverse relationship. After adjusting for children’s variables - age, physical activity in leisure time, Mediterranean diet, watching TV on a daily basis and number of hours slept per day- and parents' variables - BMI and civil status- the probability of child obesity is roughly 3% lower as income rises and with a high education background 2.2% less likely to have obese children |
| **81^130^** | **Cantallops-2012** | 4135/10-16 y/both | 2009-10 | Reported. Obesity if BMI >95-th percentile | Paternal and maternal educational level | Inverse relationship with both was observed | N/A |
|  | **SWEDEN** |  |  |  |  |  |  |
| **82^137^** | **Ekelund-2005** | 455/17 y/both | 2001-2002 | Measured. BMI, fat mass and percentage fat mass, . | Maternal educational level | Relationship varies with sex. There was a statistically significant inverse relationship between maternal educational level and fat mass, percentage of fat mass and BMI only for girls. Not for boys. For girls the correlation coefficient with fat mass and percentage of fat mass was -0.18 (p-value <0.01) and with BMI -0.16 (p-value <0.01). | N/A |
| **83^138^** | **Ekelund-2006** | 248/17 y/both | 1984/5-2001/2 | Measured. BMI. | Maternal occupation | N/A | No relationship was observed. After adjusting for weight gain in infancy (0-6 m) and during early childhood (3-6 y), sex, birth weight, gestational age and maternal BMI, the β coefficient (95% CI) was: -0.159 (-0.39, +0,0728). |
| **84^139^** | **Huus-2007** | 4242/5 y/both | 2002-2004 | Reported. Overweight and obesity; IOTF cut-off points | Maternal and paternal educational level | N/A | Relationship varies depending on indicator of SEP: an inverse relation with paternal educational level but not with maternal educational level. After adjusting for single mother, maternal age> 35 y at birth, paternal age> 37 y at birth, no sibling, (, there is an inverse relationship only between father's educational level and risk of overweight and obesity. For overweight and for obesity the ORs if father is not college (reference: university) were 1.35 (1.09-1.66) and 1.53 (1.01-2.32), respectively. |
| **85^140^** | **Huus-2008** | 3654/5 y/both | 2002-2004 | Reported. Obesity; IOTF cut-off points | Maternal and paternal educational level | N/A | No relationship was observed. To facilitate the understanding of the findings the authors of the review have changed the reference category. After adjusting for exclusive breastfeeding at least 4 months, maternal age, paternal age, single parenthood, mother born abroad, father born abroad, maternal smoking, paternal smoking the OR for maternal educational level (reference: university) was 1.28 (0.87-1.89) if not college; and the OR for paternal educational level was 1.30 (0.82-2.04) if not college. |
| **86^141^** | **Khanolkar-2012** | 1204/5-14 y/both | 2000-01 | Measured. Overweight or obesity. IOTF cut-off points | Paternal and maternal educational and occupational level | An inverse gradient was observed between measures of household SEP and prevalence of overweight/obesity. However, a statistically significant difference between extreme categories only was observed with father's and mother's education and maternal occupation but not with father's occupation. | N/A |
|  | **SWITZERLAND** |  |  |  |  |  |  |
| **87^174^** | **Buddeberg-Fischer-1996** | 1944/14-19 y/both | 1993 | Measured. Obesity if BMI>85-th and ≤ 95-th percentile and Superobesity if >95-th percentile. Swiss BMI percentiles. | Paternal occupational level | Relationship varies with sex: an inverse relationship was observed only for boys and not in girls. Literally: "The relationship between BMI weight classes and SEP was then investigated. A slightly higher percentage of underweight girls [...] and a higher percentage of overweight subjects was found in the group of low SEP (p: 0.075). For male [...] a higher percentage of obese subjects was found in the group of low SEP than in the group of higher SEP (p <0.001). " | N/A |
|  | **UNITED KINGDOM** |  |  |  |  |  |  |
| **88^82^** | **Whincup-1992** | 3842/5-7,5 y/both | 1990 | Measured. BMI. | Head of household’s occupation | No relationship was observed. | N/A |
| **89^66^** | **Duran-Tauleria-1995** | 8374/5-11 y/both | 1990-1991 | Measured. Overweight if weight for height is in the top quarter. | Maternal and paternal educational level and father's occupation | N/A | No relationship was observed. After adjusting for family size, number of weekly hours mother working outside household, number of parents in household, birth weight, presence of obese parents: in general, there is no relation between overweight and SEP indicators although children with lower maternal education level showed the lowest prevalence. |
| **90^81^** | **Van Lenthe-2001** | 509/12 y/both and 509/15 y/both | 1989-90 and 1992-93 | Measured. BMI and sum of four skinfolds thicknesses (biceps, triceps, subscapular and suprailiac)-SSF- . | Head of household’s occupation | No relationship was observed | N/A |
| **91^67^** | **Fletcher-2004** | 1208: 405 (1980); 379 (1990); 424 (2000)/11-12 y/both | 1980, 1990 and 2000 | Measured. BMI. | Parental occupation | No relationship was observed. Literally: "No sex or social class differences were found in BMI ". | N/A |
| **92^71^** | **Jebb-2004** | 1836/4-18 y/both | 1997 | Measured. Overweight and/or obesity; IOTF cut-off points | Head of household’s occupation and family income | Inverse relationship was observed with both indicators. There was a statistically significant inverse relationship between social class and obesity (p-value: 0.003) and between social class overweight plus obesity (p-value: 0.004). Literally: "There was an inverse relationship between obesity and social class, with a significantly higher prevalence of obesity in social classes IV and V than in classes I-III (6.5% vs 2.7%, p-valor: 0.003) and likewise a significant trend for overweight plus obesity (23.4% vs 17.7%, p-valor: 0.04). A significantly higher prevalence of overweight and obesity was observed in those households with annual income < 20000 pounds compared with those with ≥20000 pounds (21.1% vs 16.8%, p-valor: 0.03) but this association was not found for obesity (3.5% vs 3.6%).” | N/A |
| **93^75^** | **Saxena-2004** | 5689/2-20 y/both | 1999 | Measured. Overweight and obesity; IOTF cut-off points | Head of household’s occupation | No relationship was observed | After adjusting for age and ethnic group no relation was observed except a direct relation with obesity in girls. |
| **94^77^** | **Stamatakis-2005** | 11185/5-10 y/both | *(only since 1997) 1997; 1998-99; 2000-01 and 2002-03 | Measured. Overweight and obesity. UK 1990 BMI Reference Data cut-off points | Household income | N/A | Inverse relationship was observed. To facilitate the understanding of the findings the authors of the review have changed the reference category. After adjusting for survey year, social class according to head of household´s occupation, sex and age the OR for obesity in lower vs higher was 1.35 (1.12-1.64). |
| **95^73^** | **Ness-2006** | 5917/9,9 y/both | 1991-92 and 2001-02 | Measured. BMI and Total fat. | Parental occupation | N/A | Inverse relationship was observed. After adjusting for age and sex the average value of BMI according the different categories of social class was: 17.2 (16.9-17.6), 17.7 (17.5-17.9), 17.6 (17.4-17.7), 17.4 (17.3-17.5), 17.3 (17.2-17.5), 17.0 (16.8-17.3), with p value for trend 0.001. After adjusting for age, sex, height, height squared, the mean value of total fat according the different categories of social class was 7.1 (6.6-7.6), 7.6 (7.3-7.9), 7.4 (7.3-7.6), 7.2 (7.0-7.3) 7.0 (6.9-7.2) 6.5 (6.2-7.0), with p value <0.001. |
| **96^80^** | **Sweeting-2008** | 5667: 503 (1987); 2145 (1999); 3019 (2006)/15 y/both | 1987, 1999 and 2006 | Measured. Obesity if BMI> 95-th percentile. UK 1990 growth reference. | Head of household’s occupation | No relationship was observed | N/A |
| **97^68^** | **Gray-2008** | 1253/13-15 y/both | 1998-99 and 2003-04 | Measured. Combined overweight and obesity; IOTF cut-off points | Chief income earner of the household's occupation | No relationship was observed | After adjusting for mental health (GHQ12), age, physical activity, weekly consumption of tobacco, alcohol consumption previous week and area deprivation no relationship was observed, except in middle class in boys whose OR overweight / obesity was 1.73 (1.09-2.75) with respect high class. |
| **98^69^** | **Hawkins-2008** | 13113/3 y/both | 2003-2005 | Measured. Overweight (including obesity); IOTF cut-off points | Household income , maternal occupation and educational level | Inverse relationship was observed with maternal educational level and household income level. Household income (reference: 33,000 £ or more/y): if 0-11,000/y, OR: 1.18 (1.01- 1.37), if 11-22,000/y, OR: 1.18 (1.04-1.34), if 22-33,000/y, OR: 1.16 (1.02-1.34). Maternal educational level (reference: GCSE grades A-C or higher): if GCSE grades D-G or low, OR: 1.17 (1.04-1.31). | N/A |
| **99^76^** | **Semmler-2009** | 333/4 and 11 y/both | 1998/9-2005/6 | Measured. BMI at age 4 and at age 11. | Maternal educational level | No relationship was observed | N/A |
| **100^70^** | **Hawkins-2009** | 13188/3 y/both | 2003-2005 | Measured. Overweight (including obesity); IOTF cut-off points | Household income , maternal occupation and educational level | No relationship was observed. | N/A |
| **101^65^** | **Brophy-2009** | 17561/5 y/both | 2005-2007 | Measured. Obesity; IOTF cut-off points | Main respondent's (usually mothers') income and educational level | Relationship varies by race. Crude ORs are calculated by authors of the review from prevalence estimates. For whites/European there is an inverse relationship but not for Asians or Africans. Whites/European: for income (reference: >20,800 £ per year), if less than 10,400 £ per year, OR is 1.60; for studies (reference: university), if not qualifications, OR is 2.11, if O 'level, OR is 1.85. | Inverse relationship was observed. After adjusting for race, birth weight, enjoy making physical activity, watching TV > 3 hours per day, introducing solid foods before third month of life, smoking near the child, pre-pregnancy maternal weight > 60 kg and indoor activities, the OR for educational level were: if no qualifications or O' level versus A' level 1.81 (1.29-2.5), if no qualifications or O' level versus university, 1.49 (1.20-1.88). The OR for household income were: if <10,400 £ per year versus> 20,800, 1.19 (1-1.49) and if <10,400 £ per year versus 10,400-20,800 £ per year, 1.31 (1.07-1.63). |
| **102^72^** | **Matijasevich-2009* (only UK data)** | 6751/11 y/both | 2002-2003 | Measured. Overweight (including obesity) defined as BMI > 1 standard deviation according to WHO's growth charts of BMI-for-age and sex. | Household income and maternal educational level | For household income there was a statistically significant inverse association only in girls (p-value <0.001) and for maternal educational level there was a statistically significant inverse relationship for girls (p-value <0.001) and for boys (p-value: 0.006). | N/A |
| **103^74^** | **Pinot de Moira-2010* (only offspring)** | 1889*(only offspring)/4-9 y/both | 1991*(offspring) | Measured. BMI and combined overweight and obesity; IOTF cut-off points. | Occupation of the male head of the household | No relationship was observed. | After adjusting for sex, age, maternal and paternal BMI, birth weight, gestational age, having been breastfed and maternal smoking no association was observed. |
| **104^78^** | **Stamatakis-Zaninotto-2010** | No data/2-10 y and 11-18 y/both | 1995-2007 | Measured. Obesity; IOTF cut-off points | Head of household’s occupation | Inverse relationship was observed in both age groups. Literally: "Children aged 2-10: In relation to social class, the annual obesity prevalence was on average 0.6% (boys aged 2-10) and 1.5% (girls aged 2-10 years) higher in participants from manual than from non-manual households (figure 2). Adolescents aged 11-18: In relation to social class, the 1995-2007 annual obesity prevalence was on average 1.2% (boys 11-18 years) and 2.1% (girls 11-18 years) higher in participants from manual than from non-manual households (figure 3)". | N/A |
| **105^86^** | **Whitaker-2010** | 7078/2-15 y/both | 2001-2006 | Measured. Obesity; IOTF cut-off points | Head of household’s occupation | Inverse relationship. Crude OR is calculated by authors of the review from prevalence estimates. OR in manual vs non-manual class: 1.79. | Inverse relationship was observed. After adjusting for paternal and maternal weight, year of survey, age, sex and race, the OR en manual vs: no manual occupation was 1.54 (1.25-1.90). |
| **106^79^** | **Stamatakis-Wardle-2010** | 15271/5-10 y/both | 1997-2007 | Measured. Overweight (including obesity) and obesity; IOTF cut-off points | Household income and SEP level assesed (family income and head of household’s occupation) | Inverse relationship was observed. There is an inverse gradient since 2002-2003. Literally: "The SEP score gradient in Figure 3 also increased over time, reaching significance in 2006/7 for boys’ overweight (P<0.001) and obesity (P: 0.002), when obesity prevalence in the lower SEP score group was twice than that in the higher groups. Similar gradients in girls’ obesity prevalence were seen in 2002/3 (P<0.001), 2004/5 (P: 0.03) and 2006/7 (P:0.03)". | N/A |
| **107^83^** | **Howe-2011** | 11380/2-10 y/both | 1991/92-2001/02 | Measured. BMI | Maternal educational level | Relationship varies by gender. In boys no relationship was observed, although in 10-y group, differences in BMI between the extreme categories of maternal education was significant. In contrast, in girls an inverse gradient was observed from 7 years of age | N/A |
| **108^85^** | **Howe-2010** | 7772/9,9 y (mean)/both | 2001-03 | Measured. Total fat mass | Maternal educationa level | Inverse relationship | N/A |
| **109^84^** | **Hargreaves-2013** | 11634: 2754(1999); 847(2004);5205(2006);2828(2009)/2-16*y/both | 1999, 2004, 2006 and 2009 | Measured. Obesity if BMI≥ 95-th percentile | Occupation of the head of household or household reference person | Relationship was observed in 2006 and 2009, but not in 1999 and 2004. In 2006 and 2009, prevalence of obesity in children living in households whose head belonged to the lowest social class was 1.8 and 1.5 times higher than children living in households whose head belonged to the highest | N/A |
|  | **UNITED STATES** |  |  |  |  |  |  |
| **110^28^** | **Gerald-1994** | 77/2,5-5 y/both | 1991 | Measured. Weight for height. | Family income, and caretaker's income, educational level and Hollingshead's score (marital status, occupation and educational level) | No relationship was observed | No relationship was observed. |
| **111^57^** | **Kimm-1996** | 2379/9-10 y/only girls | 1987 | Measured. Obesity, defined as the highest quartile (>75-th percentile) of BMI of respective National Growth and Health Study population distribution | Parental educational level and household income | Relationship varies by race. There is a statistically significant inverse relationship only for white but no for black girls. White girls and parental educational level (reference: college 4 + y): if HG education or less, OR: 2.50 (1.72-3.57), if some college, OR: 1, 90 (1.30-2.80). For household income (reference: $ 40,000 or more): if <$ 10,000, OR: 2.78 (1.67-4.55), if $ 10-19999, OR: 2.39 (1.31-4, 42), if $ 20-39,999, OR: 1.31 (0.78-2.19). Black and parental educational level, if HG education or less, OR: 0.94 (0.67-1.33), if some college, OR: 1.08 (0.81-1.42). For household income: if <$ 10,000, OR: 0.72 (0.50-1.04), if $ 10-19,999, OR: 0.86 (0.58-1.27), if $ 20-39,999, OR: 1.09 (0.78-1.53). | Relationship varies with race. In white girls an inverse relationship was observed for parental educational level, but not with household income. In black girls no relationship was observed. After adjusting for number of parents, weekly hours watching TV and daily caloric intake, the OR in white girls for educational level (reference: college 4 + y) were: if less than high school, 1.89 (1.18-3.03), if some college, 1.62 (1.06-2.49). |
| **112^41^** | **Patterson-1997** | 2379/9-10 y/only girls | 1985 | Measured. Obesity, defined as BMI ≥85-th percentile of the Second National Health and Nutrition Examination Survey (NHANES II) reference population of 9- and 10-year-old girls | Parental educational level and household income | Relationship varies by race. There is a statistically significant inverse relationship only for white but no for black girls. White girls and household income (reference: ≥ $ 40,000): if <$ 20,000, OR: 2.49, if $ 20,000-39,999, OR: 1.52. For parents' educational level (reference: ≥4 y): if less than or equal to high school, OR: 2.21, if some college, OR: 1.80. | Relationship varies with race. In white girls an inverse relationship was observed for parental educational level. In black girls no relationship was observed. Adjusting for maternal age, pubertal status, number of siblings and parental occupational status in the last 12 months, the OR in white girls for parental educational level (reference: ≥ 4 y) were: if ≤ high school: 2.05, if some college, 1.87. |
| **113^58^** | **Strauss-1999** | 2913/0-8 y and 6-14 y/both | 1988-1994 | Measured. Obesity, if BMI> 95-th percentile derived from combined data of the first and second National Health and Nutrition Examination Surveys | Maternal educationa level, family income and parents' occupation | Inverse relationship was observed. For maternal education (reference: college or professional): if high school only, OR: 1.24 (0.92-1.68), if less than high school, OR: 1.47 (1.04-2.08); for parental occupation (reference: at least one parent professional / managerial): if at least one parent unprofessional, OR: 1.76 (1.15-2.67), if a parent does not work, OR: 2.36 (1.50-4.17) and for family income (reference: high): if middle, OR: 2.04 (1.21-3.44), if low , OR: 2.91 (1.66-5.08). | An inverse relationship with family income was observed. After adjusting for sex, race, marital status, maternal education, parental occupation, family income, HOME score (cognitive) and HOME score (emotional), the OR for family income (reference: high) were: if middle, 1.84 (1.01-3.34) and if low, 2.84 (1.39-5.78). |
| **114^24^** | **Crooks-1999** | 88/7-11 y/both | 1994-1995 | Measured. BMI and triceps skinfold. | Maternal and paternal educational level | No relationship was observed | N/A |
| **115^29^** | **Goodman-1999** | 15483/15,9 y (mean)/both | 1994-1995 | Reported. Obesity defined as BMI ≥95-th percentile | Household income, maternal and paternal educational level and parents' occupation | Inverse relationship was observed with education, household income and parents' occupation. | Inverse relationship was observed. After adjusting each of the indicators of SEP for other two a significant inverse relationship remains with education and income but disappears for occupation. Further after adjusting for age, sex, race/ethnicity, multiethnicity and number of parents at home a significant inverse relationship remains with education and income: the OR for education, income and occupation were 0.88 (0.79-0.98), 0.88 (0.78-0.99), and 0.88 (0.76-1.01), respectively. |
| **116^21^** | **Baughcum-2000** | 622/23-60 months/both | 1998-1999 | Measured. Overweight if weight for height ≥90-th percentile. CDC growth tables. | Maternal educational level | Inverse relationship was observed. There is a statistically significant inverse relationship (p-value: 0.001). Crude OR are calculated by authors of the review from prevalence estimates (reference: high): if low, OR: 1.53. | N/A |
| **117^38^** | **McMurray-2000** | 2389/10-16 y/both | 1995 | Measured. Overweight if BMI≥ 85-th percentile based on the NHANES II norms | Parental educational level | N/A | Inverse relationship was observed in girls. To facilitate the understanding of the findings the authors of the review have changed the reference category. After adjusting for race, daily hours watching TV, daily time spent playing videogames and level of physical activity the RR for educational level (reference: high) in girls were: if moderate, 1.66 (1.10-2.50), if low, 2.96 (1.77-4.95). In boys those RR were: if moderate, 1.66 (1.05-2.60), if low,1.31 (0.77-2.23). |
| **118^44^** | **Robinson-2001** | 792/8,4 y (female mean); 8,5 y (male mean)/both | 1996 | Measured. BMI and tricipital skinfold. | Parental educational level | Relationship varies with sex. There is only an inverse relationship in girls with BMI. Girls with BMI (BMI: r = -0.19, p-value: <0.001) and the triceps skinfold (r = -0.07, p-value: n.s.). Boys: no association (BMI: r = -0.09, triceps skinfold: r: -0.02). | N/A |
| **119^18^** | **Alaimo-2001** | 5200/2-7 y/both and 3996/8-16 y/both | 1988-1994 | Measured. Overweight: two definitions: (a) if ≥ 85-th percentile weight for height, CDC growth tables and (b) IOTF cut-off points | Family income | Relationship varies by race. Crude OR are calculated by authors of the review from prevalence estimates: inverse relationship in whites, direct in blacks and no socioeconomic gradient in Mexicans. | N/A |
| **120^49^** | **Wang-2001* (only USA data)** | 6110/6-18 y/both | 1988-1994 (*) | Measured. BMI and combined overweight and obesity defined as BMI≥ 85-th percentile, WHO-NCHS reference. | Family income | Inverse relationship was observed. Reference: high-income, if low, OR: 1.63 (1.26- 2.00) and if middle, OR: 1.25 (0.91-1.43). | Inverse relationship was observed in girls after adjusting for age and place of residence (urban vs. rural). |
| **121^40^** | **Neumark-Sztainer-2002** | 4746/11-18 y/both | 1998-1999 | Measured. At risk for overweight if BMI ≥ 85-th and < 95-th and Overweight if ≥95-th percentile. CDC growth tables | Parental educational level completed with other variables (family eligibility for public assistance, eligibility for free or reduced-cost school meals, and employment status of mother and father) | Inverse relationship was observed. In girls an inverse association with prevalence of the outcome “at risk of overweight” (trend p-value: 0.001) but not with prevalence of overweight (trend p-value: 0.168). In boys an inverse association with both: overweight (trend p-value: 0.001) and with the outcome “at risk of overweight” (trend p-value: 0.025). Crude OR are calculated by authors of the review from prevalence estimates (reference: high): Girls for overweight: if high-middle, OR: 1.88, if middle, OR: 2.60, if low-middle, OR: 2.40, if low, OR: 2.10. For the outcome “at risk of overweight”: if high-middle, OR: 1.62, if middle, OR: 2.06, if low-middle, OR: 2.40, if low, OR: 1.83. Boys: for overweight: if high-middle, OR: 1.11, if middle, OR: 1.44, if low-middle, OR: 1.46, if low, OR: 2.15. For the outcome “at risk of overweight”: if high-middle, OR: 1.09, if middle, OR: 1.09, if low-middle, OR: 1.24, if low, OR: 1.46. | N/A |
| **122^31^** | **Goodman-2003** | 15122/ 16,1 y (mean)/both | 1994-1995 | Reported. Obesity if BMI≥ 95-th percentile. CDC growth tables | Parental educational level and household income | Inverse relationship was observed. For overall sample there is an inverse relationship with both SEP indicators. Household income (reference: quintile 5 -Q5-): if Q1, OR = 2.04 (1.71 -2.44), if Q2, OR: 1.79 (1.49-2.12), if Q3, OR: 1.88 (1.59-2.22), if Q4, OR: 1.33 (1.71-2.44). Parental educational level (reference: professional degree): if <high school, OR: 2.26 (1.82-2.81), if high school, OR: 2.17 (1.80-2.64) and if> high school and <college, OR: 1.76 (1.45-2.14), if college graduate, OR: 1.39 (1.12-1.93). By sex and race this relationship disappears for nonwhite males and persists for white and non-white girls and white boys. Household income: overall sample (p-value <0.001) for white boys and girls (p-value <0.001) and non-white girls (p-value: 0.04). Educational level: overall sample (p-value <0.001) for white boys and girls (p-value <0.001) and non-white girls (p-value: 0.01). | N/A |
| **123^19^** | **Anderson-2003** | 16650/3-11 y/both | 1996-2001 | Measured. Overweight if BMI>95-th percentile. CDC charts | Household income and maternal educational level | Inverse relationship was observed. Crude OR are calculated by authors of the review from prevalence estimates. Household income level (reference: fourth quartile): if first quartile, OR: 1.52, if second, OR: 1.34, if third, OR: 1.43. Maternal educational level (reference: some college or more): if less than high school degree, OR: 1.40, if high school degree, OR: 1.14. | Inverse relationship was observed. After adjusting for average number of weekly hours of maternal work (since birth of child), number of weeks worked by the mother since birth of child, race, maternal score for Armed Forces Qualification Test, to be or not the firstborn, number of siblings, breastfed, presence of maternal overweight or obesity, maternal marital status, overweight risk decreases by 0.4% for every additional year of maternal school, and by 0.2% for each increment of $ 10,000 in family income. |
| **124^32^** | **Gordon-Larsen-2003** | 13113/12-20 y/both | 1996 | Measured. Overweight if BMI≥85-th percentile. 2000 CDC growth charts | Parental educational level and household income | Relationship varies depending on sex and race. Boys: no relationship was observed. Girls: an inverse gradient with household income and parental educational level in White and Hispanic but not in African or Asian girls. | N/A |
| **125^33^** | **Haas-2003** | 3775/6-17 y/both | 1996 | Reported. Overweight if BMI≥ 95-th percentile. CDC growth charts | Parental educational level and household income | Inverse relationship was observed. Crude OR are calculated by authors of the review from prevalence estimates. Children (6-11 y): parental educational level (reference: >12 y): if ≤12 y, OR: 1.63. Household income (reference: ≥ 125%): if < 125%, OR: 1.88. Adolescents (12-17 y): also inverse relationship. Parental educational level: if ≤12 y, OR: 1.48. Household income: if < 125%, OR: 1.13. | Results vary by age group and indicator of SEP. After adjusting for sex, age, race, country of child birth, parenthood, U.S. region of residence, currently receiving Aid to Families with Dependent Children program (AFDC), and health insurance status, the OR in children aged 6 to 11 years for parental educational level (reference: if > 12 y) was: if ≤ 12 y, 1.38 (1.05-1.82). However no relationship was observed for household income. In adolescents aged 12 to 17 years the OR for household income (reference: if ≥125%) was if income < 125%, 0.53 (0.34-0.83). However no relationship was observed for educational level. |
| **126^59^** | **Lumeng-2003** | 755/8-11 y/both | 1998 | Measured (80%) and reported (20%). Overweight if BMI≥95-th percentile. 2000 CDC growth charts | Maternal educational level and Family poverty status (total family income, family size, and state of residence) | Relationship varies depending on SEP indicator. An inverse relationship was observed with maternal education level and no with poverty level. Maternal educational level (reference: "high school" completed): if less than high school diploma, OR: 2.41 (1.09 -5.31). Poverty level (reference: non-poverty): if in poverty, OR: 0.64 (0.25-1.65). | No relationship was observed after adjusting for BPI (Behavior Problems Index) score >90-th percentile, sex, race, medication for behavior intake, academic grade retention, HOME-SF cognitive stimulation score, daily hours TV watching, maternal obesity, maternal smoking, single mother and maternal depressive symptoms. |
| **127^30^** | **Goodman-2003** | 1491/15,2 y (mean)/both | 2001-2002 | Measured. Overweight if BMI ≥95-th percentile. CDC growth charts | Parental educational level and household income | Results change by sex and race. There is only a statistically significant inverse relationship in white girls. Parental educational: by decreasing a level increases the risk by 43% (OR: 1.43; 1.08-1.89). Household income: by decreasing a level increases the risk by 25% (OR: 1.25; 1.00-1.56). | N/A |
| **128^25^** | **Datar-2004** | 9751/no age (kindergarten's and grade's 1)/both | 1998-2000 | Measured. Owerweight if BMI ≥95-th percentile | Maternal educational level and family income | Inverse relationship was observed. Literally: “…children whose mothers had an educational level of a high school diploma or less, and children from low-income families were significantly more likely to be overweight in kindergarten as well as first grade”. Crude OR are calculated by authors of the review from prevalence estimates. For Fall Kindergarten: maternal educational level (reference: bachelor): if less than high school, OR: 1.66, if high school, OR: 1.53, if some college, OR: 1.29. Family income (reference: ≥$ 75,000): if <$15,000, OR 1.60, if 15-24,999, OR: 1.87, if 25-34,999, OR: 2.10; if 35-49,999, OR: 1, 43, if $50-74,999, OR: 1.34. For Grade 1: maternal educational level: if less than high school, OR: 1.93, if high school, OR: 1.59, if some college, OR: 1.33. Family income: if <$15,000, OR: 1.77, if $15-24,999, OR: 1.97, if $25-34,999, OR: 1.83, if $35-49,999, OR: 1.45, if $50-74,999, OR: 1.22. | N/A |
| **129^35^** | **Hofferth-2005** | 1449/6-12 y/both | 1997 | Reported. BMI and overweight (if BMI ≥95-th percentile). 2000 CDC growth charts | Family income | No relationship was observed. Literally: “Overall there is no linear association between the income to poverty ratio and overweight. The linear variable was never significantly associated with overweight or BMI (not shown). However, we hypothesized that the relationship might not be linear". Children in families with incomes between 100 and 300% of the poverty were more likely to be overweight (22-24%) than children from families with incomes below 100 % of the poverty line (11.3 %) or children in families with incomes of 300 % of poverty or higher (11.5 %). The results for BMI are similar. Prevalence of overweight and BMI mean value increase with levels of household income: 11.3 %, 24.7 %, 23.9 %, 22.5 %, 11.5 % and 17.8, 19.8, 18.8, 19 and 18 kg/m2 respectively. | Nonlinear relationship was observed. The poorest and the richest are those with a lower frequency of overweight and smaller BMI after adjusting for other household incomes and expenditures, breakfast and / or school lunch, race, parental studies, age, number of children in household, age of head of household, sex, low birth weight, parental employment status, parenthood. |
| **130^23^** | **Classen-2005** | 4980/2-18 y/both | 1986-2002 | Measured. Overweight if BMI ≥85-th percentile and obesity if ≥95-th percentile. 2000 CDC growth charts | Maternal educational level and income | N/A | An inverse relationship with maternal education level was observed. |
| **131^42^** | **Rhee-2006** | 872/7 y/both | 1998-1999 | Measured. Overweight if BMI ≥95-th percentile. National Center for Health Statistics growth curves | Maternal educational level and family income | N/A | After adjusting for race, maternal expectations on child self-control and maternal sensitivity about the child's needs for income / needs ratio, an inverse relationship was observed. When added to the model sex, maternal education, single parenthood, score Child Behavior Problems Checklist, the relationship disappeared. |
| **132^50^** | **Wang-2006** | 30417: 6555 [NHAES I (1971-5)]; 6741 [NHAES II (1976-80)]; 9731 [NHAES III (1988-94)]; 7390 [NHAES 1999-2002]/2-18 y/both | 1971-2002 | Measured. BMI and overweight (if BMI ≥95-th percentile). 2000 CDC growth charts | Household income | Relationship differs by survey year, age group, sex and race (Figures 1 and 2 and Tables 2 and 3). The results for the outcome "at risk of overweight " are similar to those of overweight and therefore only results for overweight are presented. Literally: "the trends in SEP-related disparity patterns varied across age, sex, and ethnic groups. In boys aged 2–9 y, a significant reverse association between family income and overweight appeared in 1999–2002 (Figure 1). In girls aged 2–9 y, none of the associations were significant. A strong reverse association was observed in adolescent boys aged 10–18 y in NHANES III (1988 –1994), but it became statistically nonsignificant by 1999–2002. In adolescent girls, the reverse association between family income and overweight remained significant between 1976 and 1980 and between 1999 and 2002, but the ratio in the prevalence of overweight in the low-SEP group compared with the high-SEP group decreased from 3.1 in NHANES III (1988–1994) to 1.6 in 1999–2002. Ethnic differences in the changing trends in economic disparities (ie, the association) are showed in Figure 2. Considerable differences in the association between SEP and overweight across ethnic groups were observed. In white adolescent boys and girls, there was a significant reverse association in NHANES III (1988 –1994). In contrast, black girls with a high SEP had a higher prevalence than did their low- and medium-SEP counterparts in 1988–1994 and 1999–2002. For Mexican Americans, the study sample was only large enough with NHANES III (1988 –1994) to allow us to examine the trends. There were no consistent patterns”. | N/A |
| **133^45^** | **Rose-2006** | 16889/74,7 months (mean)/both | 1999 | Measured. Overweight if BMI ≥95-th percentile. 2000 CDC growth charts | Maternal educational level and household income by Poverty Index Ratio (PIR) | Inverse relationship was observed. Maternal educational level (reference: college graduate): if less than high school graduate, OR: 1.60 (1.29-1.99), if high school graduate, OR: 1.47 (1.23-1.79). Poverty Index Ratio –PIR_ (Reference: ≥ 5): if PIR <1, OR: 1.20 (0.94-1.54), if PIR between 1 and 1.85, OR 1.35 (1.11-1.65), if PIR between 1.85 to 3, OR: 1.27 (1.02-1.58), if PIR between 3 and 5, OR: 1.08 (0.87-1.35). | N/A |
| **134^39^** | **Miech-2006** | 4748*(only NHAES 1999-2004)/12-17 y/both | 1999-2004 | Measured. Overweight if BMI ≥95-th percentile. 2000 CDC growth charts | Household income by Poverty income ratio | Results vary according to race. In group 15-17 y there is a clear inverse relationship in boys and girls and in white and black race. In group 12-14 y: the same except a direct relationship in blacks. | N/A |
| **135^26^** | **Delva-2006** | 62156/no age (8th's grade)/both; 64899/no age (10th's grade)/both and 35107/no age (12th's grade)/both | 1986-2003 | Reported. Overweight if BMI ≥85-th percentile. CDC growth charts | Parental educational level | Inverse relationship was observed, although this relationship was not quantified. | N/A |
| **136^51^** | **Whitaker-2007** | 2412/3 y/both | 2001-2003 | Measured. Obesity if BMI ≥95-th percentile. 2000 CDC growth charts | Maternal educational level and household income | Relationship varies with indicator of SEP. An inverse relationship with maternal educational level (p-value: 0.04) and no relation with household income (p-value: 0.12). | N/A |
| **137^27^** | **Delva-2007** | 39011/no age (8th's and 10th's grades)/both | 1998-2003 | Reported. Being ≥85-th percentile. CDC growth charts | Parental educational level | Inverse relationship was observed. Crude OR are calculated by authors of the review from prevalence estimates. White girls (reference: high SEP): if middle, OR: 1.59 and if low, OR: 2.70. Black: if middle, OR: 1.53 and if low, OR: 1.23. Hispanics: if half OR: 1.44 and if low, OR: 1.91. White boys: if middle, OR: 1.64 and if low, OR: 2.23. Black: if middle, OR: 1.14 and if low, OR: 1.09. Hispanics: if middle, OR: 1.30 and if low, OR: 1.40. Greatest inequalities in obesity are seen among whites and smallest among blacks. | An inverse relationship was observed. After adjusting for year of the survey, school grade level, population density, geographic region, lifestyles (frequency of intake, of sleep, of exercise and watching TV), family / parenting (number of hours alone, living with two parents or not, mother's employment status), the ORs for categories of SEP and race (reference category: withes of the higher SEP) in girls were: if medium in whites, 1.40 (1.24-1.59), if low in whites 2.11 (1.77-2.51); if high in blacks, 1.98 (1.52-2.58), if medium in blacks, 2.72 (2.28-3.26), if low in blacks, 2.09 (1.59-2.76); if high in Hispanics, 1.54 (1.01-2.34), if medium in Hispanics, 2.06 (1.65-2.58), if low in Hispanics, OR: 2.65 (2.08-3.36). The ORs for categories of SEP and race (reference category: withes of the higher SEP) in boys were: if medium in whites, 1.48 (1.33-1.63), if low in whites 1.75 (1.49-2.05), if high in blacks, 1.41 (1.11-1.79), if medium in blacks, 1.53 (1.29-1.82), if low in blacks, 1.33 (0.96-1.84), if high in Hispanics, 1.63 (1.19-2.23), if medium in Hispanics, 2.03 (1.71-2.42), if low in Hispanics, 2.14 (1.75-2.62). |
| **138^34^** | **Hernandez-Valero-2007** | 438/5-18 y/both | 2001-2003 | Reported. At risk of overweight plus overweight (if BMI≥85-th percentile) and Overweight only (if >95-th percentile). | Maternal educational level | N/A | An inverse relationship was observed for overweight. After adjusting for age, sex and physical activity level the OR for overweight (reference: studies ≥ high school) was: if < high school, OR: 2.04 (1.14-3.57). |
| **139^17^** | **Ahn-2008** | 4010/12-17 y/both | 2003-2004 | Reported. AROW (overweight plus at risk of overweight) if BMI ≥85-th percentile. CDC growth charts | Parents' educational level and household income (as percentage of federal poverty threshold) | Inverse relationship was observed. Boys: parental educational level (reference: college or more): if less than high school graduate, OR: 2.67 (1.86-3.84), if high school graduate, OR: 1.39 (0.96-2.03), if some college, OR: 1.91 (1.33-2.75). Household income (reference: ≥ 100%): if <100%, OR: 1.44 (1.03-2.03). Girls: parental educational level: if less than high school graduate, OR: 2.95 (1.77-4.94), if high school graduate, OR: 2.95 (1.81-4.81) and if some college, OR: 2.06 (1.25-3.40). Household income: if <100%, OR: 1.79 (1.25-2.56). | Inverse relationship was observed with parental educational level. After adjusting for age, race, length of stay in USA, dietary habit score, performing regular moderate / vigorous physical activity, soft drink vending machine at school, park or playground within walking distance of home, the ORs for parental educational level (reference: college or more) in boys were: if less than high school graduate, 2.07 (1.28-3.35), if high school graduate, 1.16 (0.79-1.71), if some college, 1.72 (1.18-2.51); the ORs for household income (reference: ≥ 100%) was: if <100%, 1.05 (0.72-1.58). The ORs for parental educational level (reference: college or more) in boys were: if less than high school graduate, 2.00 (1.05-3.80), if high school graduate, 2.33 (1.39-3.90) and if some college, 1.71 (1.04-2.81); the ORs for household income (reference: ≥ 100%) was: if <100%, OR: 1.28 (0.79-2.08). |
| **140^37^** | **Lutfiyya-2008** | 62976/5-18 y/both | 2003-2004 | Reported. Overweight (if BMI ≥85-th and <95-th percentile) and/or obesity (if ≥95-th percentile) | Household income (as percentage of federal poverty level) | N/A | Inverse relationship was observed. After adjusting for sex, preventive care during past 12 months, father meeting at least recommended levels of moderate physical activity, mother meeting at least recommended levels of moderate physical activity, child meeting at least recommended levels of moderate physical activity, sports team participation, child’s non school computer use, child’s TV use, there is an inverse relationship was observed between overweight/Obesity and household income in Whites, African Americans and Hispanics. |
| **141^46^** | **Singh-2008** | 46707/10-17 y/both | 2003-2004 | Reported. Obesity if BMI ≥95-th percentile. 2000 CDC growth charts | Household or parental education level and Household poverty status (ratio of family income to poverty threshold) | N/A | Inverse relationship was observed. After adjusting for age, sex, race / ethnicity, household composition, state of residence, place of residence, language spoken, neighborhood safety, social capital, daily hours of watching TV, recreational computer use, physical activity and participation in extrascholar sports, the ORs for parental or household educational level (reference; ≥ 13 y) were if <12 y, 1.41 (1.11-1.79), if 12 y, 1.39 (1.25-1.55). The Ors for household poverty level (reference: ≥400%) were: if <100%, 1.69 (1.41-2.03), if 100-150%, 1.63 (1.35-1.97), if 150-200%, 1.52 (1.26-1.83), if 200-300%, 1.40 (1.20-1.62), if 300 - 400%, 1.27 (1.09-1.48). |
| **142^60^** | **Singh-2008** | 46707/10-17 y/both | 2003-2004 | Reported. Obesity if BMI ≥95-th percentile. 2000 CDC growth charts | Household or parental education level and Household poverty status (ratio of family income to poverty threshold) | Inverse relationship was observed. Boys: educational level (reference: ≥13 y): if <12 y, OR: 1.85 (1.40-2.46), if 12 y: 1, 67 (1.47-1.91). Household poverty status (reference: ≥ 400%): If <100%, OR: 2.64 (2.14-3.27), if 100-200%, OR: 2.15 (1.78-2.59), if 200-400%, OR: 1.58 (1.33-1.88). Girls: educational level: if <12 y, OR: 2.80 (2.08-3.77), if 12 y, OR: 2.13 (1.81-2.50). Household poverty status: if <100%, OR: 3.09 (2.43-3.93), if 100-200%, OR: 2.44 (1.94-3.05), if 200-400%, OR: 1.53 (1.24-1.89). | Inverse relationship was observed. After adjusting for race, place of residence, social capital index, perceived neighborhood safety, daily number of hours to watch TV, daily number of hours of computer use purposes not homework, weekly physical activity, and participation in sports, the ORs for educational level (reference: ≥13 y) in boys were: if <12 y, 1.22 (0.88-1.69), if 12 y, 1.33 (1.16-1.53). The ORs for household poverty status (reference: ≥ 400%) were: if <100%, 1.78 (1.41- 2.24), if 100-200%, 1.60 (1.32-1.92), if 200-400%, 1.38 (1.19-1.61). The ORs for educational level in girls were: if <12 y, 1.74 (1.23-2.48), if 12 y, 1.56 (1.30-1.86). The ORs for household poverty status were: if <100%, 1.55 (1.17-2.07), if 100-200%, 1.50 (1.16-1.94), if 200-400 %, 1.22 (0.99-1.51). |
| **143^43^** | **Richmond-2008** | 17007/12-19 y/both | 1994-1996 | Reported. BMI. | Maternal educational level and household income (as percentage of poverty level) | N/A | Relationship varies by sex. Maternal education and household income showed no association with BMI in boys, but they showed a significant inverse relationship in girls. |
| **144^62^** | **Liu-2008** | 47757 /10-17 y/both | 2003 | Reported. Overweight if BMI ≥95-th percentile. 2000 CDC growth charts | Household income (as household poverty status) and parental educational level | Inverse relationship was observed. Crude OR are calculated by authors of the review from prevalence estimates. Household poverty status (reference: ≥ 400%): if <100%, OR: 2.95, if 100-199%, OR: 2.33, if 200-399%, OR: 1.57. Parental educational level (reference: > 12 y): if <12 y, OR: 2.09, if 12 y, OR: 1.80. | Inverse relationship was observed. After adjusting for place of residence, physical activity, sex, age, race, use of electronic media, child health, number of children in the family, perceived neighborhood safety, parental physical activity and region of residence, the ORs for household poverty status (reference: ≥ 400%) were: if <100%, 1.68 (1.40-2.02), if 100-199% , 1.58 (1.35-1.85), if 200-399%, 1.34 (1.18-1.52). The ORs for parental educational level (reference: > 12 y) were: if < 12 y, 1.19 (0.93-1.53) and if 12 y, 1.30 (1.17-1.46). |
| **145^48^** | **Voorhees-2009** | 1554/11-12 y/only girls | 2003 | Measured. BMI. | Parental educational level | Results vary by race. There is a statistically significant inverse relationship (p-value: <0.10) in Hispanic and other races but not in white or black race. | No relationship was observed after adjusting for parental employment status, use of free or subsidized meals, race, school’s deprivation and area of residence. |
| **146^47^** | **Skelton-2009** | 12384/2-19 y/both | 1976-80; 1988-94; 1999-2004 | Measured. Obesity (if BMI ≥95-th percentile) and morbid obesity (if BMI ≥40 kg/m^2^). CDC growth charts. | Household income (as Poverty Income Ratio) | Inverse relationship was observed. Crude OR are calculated by authors of the review from prevalence estimates. Obesity (2-19 y): Poverty income ratio (reference:> 3): if <1, OR: 1.39 and if 1-3 OR: 1.28 (p-value <0.001). Morbid obesity (12-19 y): if <1, OR: 2.53 and if 1-3 OR: 1.38 (p-value: 0.02). | N/A |
| **147^22^** | **Butte-2009** | 1030/4-19 y/both | 2000-2004 | No information about measured or reported. Overweight if BMI ≥95-th percentile. CDC growth charts | Household income, maternal and paternal educational level | There is an inverse relationship only with paternal educational level. Literally: "Risk decreased with increasing years of paternal education". | No relationship was observed. |
| **148^61^** | **Singh-2009** | 46707/10-17 y/both | 2003-2004 | Reported. Overweight (if BMI ≥85-th percentile) and obesity (if ≥95-th percentile). 2000 CDC growth charts | Household income (as household poverty status) and household or parental educational level | Inverse relationship was observed. Obesity: parental educational level (reference: ≥ 13 y): if <12 y, OR: 2.22 (1.80-2.72), if 12 y, OR: 1.84 (1.67-2.04); Household income (reference: ≥ 400%): if below 100%, OR: 2.82 (2.39-3.31), if 100-200%, OR: 2.26 (1.96-2.60), if 200-400%, OR: 1.56 (1.36-1.79). Overweight: parental educational level: if <12 y, OR: 1.93 (1.62-2.31), if 12 y, OR: 1.69 (1.55-1.83): Household income: if below 100%, OR: 2.26 (2.00-2.56), if 100-200%, OR: 1.99 (1.80-2.21), if 200-400%, OR: 1.38 (1.26-1.51). | An inverse relationship was observed. After adjusting for immigrant status, region of residence, perceived neighborhood safety, daily hours of watching TV, daily hours of computer use for not educational purposes and weekly physical activity) the ORs for parental educational level (reference: ≥ 13 y) in obesity were: if <12 y, 1.30 (1.01-1.67), if 12 y, 1.32 (1.18-1.48). The ORs for household poverty status (reference: ≥ 400%) in obesity were: if below 100%, 1.68 (1.40-2.02), if 100-200%, 1.57 (1.34-1.83), if 200-400%, 1.33 (1.17-1.51). The ORs for parental educational level (reference: ≥ 13 y) in overweight were: if <12 y, 1.21 (0.98-1.48), if 12 y 1.29 (1.18-1.41). The ORs for household poverty status in overweight were: if below 100%, 1.48 (1.27-1.73), if 100-200%, 1.48 (1.32-1.66), if 200-400% , 1.20 (1.10-1.32). |
| **149^63^** | **Morgenstern-2009* (only USA)** | 4473/12-16 y/both | No data | Reported. At risk for overweight (if ≥85-th and ≤95-th percentile), Overweight (if >95-th percentile) and BMI. 2000 CDC growth charts. | SEP level assesed (parental education and family income) | Inverse relationship was observed. There is a statistically significant inverse relationship between SEP and weight status (p <0.001). | N/A |
| **150^20^** | **Anderson-2010** | 8550/4 y/both | 2005 | Measured. Obesity if BMI ≥ 95-th percentile. 2000 CDC growth chart | Maternal educational level and household income (as household income to poverty ratio) | Inverse relationship was observed: Crude OR are calculated by authors of the review from prevalence estimates. Maternal educational level (reference: bachelor's degree or higher): if some college/vocational or technical degree, OR: 1.42, if high school diploma/general equivalency diploma, OR: 1.64, if less than high school, OR: 2.08. Household income (reference: > 3): if <0.50, OR 1.31, if 0.50-0.99, OR: 1.59, if 1 to 1.85, OR: 1.51, if 1.86 to 3, OR: 1.07. | N/A |
| **151^64^** | **Singh-2010** | 90808: 46707 (2003) and 44101 (2007)/10-17 y/both | 2003-2004 and 2007-2008 | Reported. Overweight (if BMI ≥85-th) and obesity (if BMI≥P95-th percentile). 2000 CDC growth chart | Household or parental educational level and household income (as Household poverty status) | Inverse relationship was observed. Crude OR are calculated by authors of the review from prevalence estimates. Obesity: educational level (reference: ≥16 y of education): Year 2007: if <12 y, OR: 3.12, if 12 y, OR 2.11, if 13-15 y, OR: 1.84. Year 2003: 2.19, 1.90 and 1.56, respectively. Household income (reference: ≥400 %): Year 2007: if <100 %, OR: 2.75, if 100-199 %, OR: 2.12, if 200-399 %, OR: 1.46. Year 2003: 2.38, 2.00 and 1.48, respectively. Overweight: educational level: Year 2007: if <12 y, OR: 2.08, if 12 y, OR: 1.68, if 13-15 y, OR: 1.50. Year 2003: 1.68, 1.54 and 1.35, respectively. Household income: Year 2007: if <100 %, OR: 2.02, if 100-199%, OR: 1.70, if 200-399 %, OR: 1.36. Year 2003, 1.74, 1.61 and, 1.26, respectively. | An inverse relationship was observed. In 2007, after adjusting for age, sex, race, household composition, place of residence, household primary language, household employment status, perceived neighborhood safety, geographic region, number of daily hours watching TV, number of daily hours use of computer not for homework, physical activity. The ORs for educational level (reference ≥ 16 y) in obesity were: if < 12 y, 2.52 (1.89- 3.36), if 12 y, 1.91 (1.57-2.33), if 13-15 y, 1.69 (1.41-2.01). The ORs for household income (reference: ≥ 400 %) were: if < 100 %, 2.23 (1.67-2.98),), if 100-199 %, 1.87 (1.48-2.38) and if 200-399 %, 1.34 (1.09-1.66). The ORs for educational level in overweight were 2.11 (1.64-2.71), 1.75 (1.49-2.06) and 1.52 (1.33-1.74), respectively. The ORs for household income in overweight were 2.11 (1.69-2.62), 1.73 (1.45-2.06), 1.36 (1.18-1.58), respectively. Similar findings were observed in 2003. |
| **152^36^** | **Li-2010** | 62880/6-11 y and 12-17 y/both | 2003-2004 | Reported. BMI. | Parental educational level and household income (as Household poverty level) | N/A | An inverse relationship was observed. Literally: "Parents’ education level is negatively associated with the child’s BMI; parents with a high school education level are associated with an average reduction in BMI of 0.517 in the child’s BMI value compared to children whose parents’ education level is lower than high school. Parents with college and above education level are linked to a 1.279 reduction in the child’s BMI value". If household income is below 300% of poverty level The BMI increases 0.334 with respect to those above. The probability of overweight decreases on average 0.069 for those with education level high school versus those who do not have and 0.248 in those with level college and above versus those who do not. |
| **153^53^** | **Ogden-2010** | No data/2-19 y/both | 2005-2008 | Measured. Obesity if BMI≥P95-th percentile. 2000 CDC growth chart | Household income (as Poverty Income Ratio) and Household head educational level | Childhood obesity prevalence decreases as income increases and as the education of head of household increases, but the relationship is not consistent across race and ethnicity groups. Specifically, the relationship is significant only for Non Hispanic-white children in the case of income and for Non Hispanic-white and Non Hispanic-black girls in the case of education. | N/A |
| **154^54^** | **Khullar-2011** | 1298/12-19 y/both | 2001-2002 | Measured. BMI | Parental educational level | Youth from families with highly educated parent(s) had lower BMI z-scores | Addition of dispositional optimism to the regression model caused an approximately 10% attenuation of the parent education and BMI z score relationship |
| **155^55^** | **Cardel-2012** | 267/7-12 y/both | 2005-2008 | Measured. Trunk fat, Total abdominal adipose tissue, BMI. | Hollingshead 4-factor index (educational attainment and occupational prestige for working parents) | N/A | SEP was inversely associated with child adiposity (β=0–.28, p<.0001), where a 1 SD increase in SEP resulted in a −0.28 SD shift in child adiposity, after adjusting for all other predictors (parental restriction, sex, pubertal status, african admixured, european admixured and parental pressure to eat). |
| **156^52^** | **Rossen-2012** | 7066: 3805 and 2361/2-18 y/both | 2001/02 and 2009/10 | Measured. Obesity if BMI≥P95-th percentile. 2000 CDC growth chart | Household income by Poverty Income Ratio | Inverse relationship. With respect to the reference group of income (> 400% of federal poverty level) the prevalence of obesity in the group with lowest income (0 to 100% of federal poverty level) was 1.8 times greater in both period of analysis, 2001/02 and 2009/10. | N/A |
| **157^56^** | **Pardo-Crespo_2013** | 746/1-17 y/both | 2006 | Reported. Overweight if BMI≥P95-th percentile. | Parental educational level and household income | Relationship depends on SEP indicator: inverse relationship with household income and absence with parental educational level | N/A |
|  | **FROM SEVERAL COUNTRIES** |  |  |  |  |  |  |
| **158^168^** | **Bammann-2012** | 7988/2-9 y/both | 2007-2008 | Measured. Overweight (including obesity); IOTF cut-off points | Parental educational level, parental occupational level and household income | Inverse relationship. Study carried out in samples of children from Belgium, Germany, Italy, Spain and Sweden (apart from other countries that we are the subject of this review). The relationship of the three indicators of socioeconomic position showed an inverse gradient with overweight, including obesity, although the magnitude of the relationship varies between countries and between indicators of socioeconomic position. | N/A |
|  | *(*): analyzed only the results of one group discarding the rest. (**): Quetelet Index or Body Mass Index (BMI) calculated ( weight in kg/height^2^ in meters) and percentiles and cut-offs are related to age and sex* | | | | | | |
|  | *SEP: socioeconomic position; WHO: World Health Organization; CDC: Centers for Disease Control and Prevention; NCHS: National Center for Health Statistics; P: percentile; OR: odds ratio; IOTF: International Obesity Task Force; PRR prevalence rate ratio.* | | | | | | |
|  |  | | | | | | |
